# Application of biomaterials in the eradication of *Helicobacter pylori*: A bibliometric analysis and overview

**DOI:** 10.3389/fmicb.2023.1081271

**Published:** 2023-03-16

**Authors:** Chunxi Shu, Zhou Xu, Cong He, Xinbo Xu, Yanan Zhou, Baihui Cai, Yin Zhu

**Affiliations:** ^1^Department of Gastroenterology, Digestive Disease Hospital, The First Affiliated Hospital of Nanchang University, Nanchang, Jiangxi, China; ^2^The Second Clinical Medical College of Nanchang University, Nanchang, Jiangxi, China

**Keywords:** *Helicobacter pylori*, biomaterials, eradication, nanoparticles, drug delivery, drug resistance

## Abstract

*Helicobacter pylori* is a prominent cause of gastritis, peptic ulcer, and gastric cancer. It is naturally colonized on the surface of the mucus layer and mucosal epithelial cells of the gastric sinus, surrounded not only by mucus layer with high viscosity that prevents the contact of drug molecules with bacteria but also by multitudinous gastric acid and pepsin, inactivating the antibacterial drug. With high-performance biocompatibility and biological specificity, biomaterials emerge as promising prospects closely associated with *H. pylori* eradication recently. Aiming to thoroughly summarize the progressing research in this field, we have screened 101 publications from the web of science database and then a bibliometric investigation was performed on the research trends of the application of biomaterials in eradicating *H. pylori* over the last decade utilizing VOSviewer and CiteSpace to establish the relationship between the publications, countries, institutions, authors, and most relevant topics. Keyword analysis illustrates biomaterials including nanoparticles (NPs), metallic materials, liposomes, and polymers are employed most frequently. Depending on their constituent materials and characterized structures, biomaterials exhibit diverse prospects in eradicating *H. pylori* regarding extending drug delivery time, avoiding drug inactivation, target response, and addressing drug resistance. Furthermore, we overviewed the challenges and forthcoming research perspective of high-performance biomaterials in *H. pylori* eradication based on recent studies.

## 1. Introduction

*Helicobacter pylori* is a pathogenic Gram-negative spiral-shaped bacteria that infects approximately 4.4 billion people worldwide, which is therefore considered to be one of the most prevalent infections worldwide (Rajinikanth et al., 2007; Hooi et al., 2017; Reshetnyak and Reshetnyak, 2017). Among those with the disease, *H. pylori* primarily generally cause chronic gastritis and lead to gastric ulcers and gastric atrophy, furthermore, induces intestinal metaplasia and, in severe cases, gastric cancer (Capurro et al., 2019). Such strong infectivity and pathogenicity make *H. pylori* recognized as Class 1 carcinogen and a major risk factor for the development of gastric cancer, which is highly thought of as the third leading cause of death worldwide (Mera et al., 2018). The pioneering *H. pylori* eradication regimen was the standard triple therapy consisting of proton pump inhibitors (PPI), amoxicillin, and clarithromycin or metronidazole proposed by the European Maastricht V/Florence consensus report (Malfertheiner et al., 2007). However, the emergence of resistant strains of metronidazole and clarithromycin has led to a steady decline in the eradication rate of standard triple therapy. For this purpose, recently a new strategy has been implemented in various regions of the world, namely quadruple therapy containing bismuth agent (bismuth agent +PPI+ two antibiotics) is highly recommended when high resistance of clarithromycin and metronidazole occurs (Fallone et al., 2016; Malfertheiner et al., 2017). Nevertheless, eradication of established *H. pylori* infection *in vivo* is challenging due to several factors concerning the duration of drug administration, primary antibiotic resistance, and stability of gastric acid secretion therapy (Praditya et al., 2019). Conventional medicine necessitates frequent administration because of its short half-life in the gastric mucus, thus causing non-negligible side effects regarding the mucosal microbiome (Coker et al., 2018). Given these factors, a reasonable approach to promote therapeutic outcomes is to develop the ability to deliver anti-suitable drugs in the gastric niche, while considering the stability and compatibility of therapeutic agents in an acidic environment. Apparently, owing to their unique potential regarding beneficial biocompatibility and bioactivity, advanced biomaterials are rapidly becoming a promising research trend in the field (de Souza et al., 2021).

Biomaterials are currently defined as substances that have been designed to take a form that, alone or as part of a complex system, is designed to guide the process of any therapeutic or diagnostic process by controlling the interaction with the components of the living system (Zhang et al., 2020; Butkovich et al., 2021). Biomaterials are generally classified into three categories: organic, inorganic, and bio-based materials. Among them, bio-based materials are mainly derived from cells, bacteria, and viruses, such as protein-based nano systems and outer membrane vesicles. In addition, according to their sources, biomaterials can be divided into natural and synthetic materials (Han et al., 2022). Natural biomaterials have been utilized for a long time due to their superior biocompatibility, biodegradability, low toxicity, and hypoallergenic, and the degradation products yielded are less cytotoxic, thus metabolized more easily by host tissues (Zhu et al., 2021; Han et al., 2022). Nowadays, biomaterials have achieved encouraging prospects in various fields, as well as increasingly becoming a new hotspot in the treatment of *H. pylori* (de Souza et al., 2021). Combining drugs with advanced biomaterials systems not only enables specific response delivery to the *H. pylori* parasite site but also prolongs the release rate of drugs at the target site (Darroudi et al., 2021). The application of biomaterials for the eradication of high drug resistance of *H. pylori* has become a new research trend. Here, bibliometrics and visual analysis are primarily adopted in the “quantitative analysis” section to generally explore the characteristics of studies on eradicating *H. pylori* with biomaterials over the past decade. Additionally, the main research topics and emerging trends are reviewed in the “main text” section based on the bibliometric analysis, and the potential challenges and forthcoming prospects of *H. pylori* eradication by biomaterials are discussed insightfully.

## 2. Quantitative analysis

Focused on the research trends in biomaterials for *H. pylori* eradication, this study employs bibliometric analysis to achieve visualization of the related topic. The bibliometric analysis allows not only quantitative and qualitative evaluation of publications but also the prediction of trends in a research field. It makes it possible to present the most influential research results and provide a theoretical basis for further research quickly and accurately (Ouyang et al., 2021). Through a decade of relevant bibliometric analysis, we overviewed the research progress of treating *H. pylori* with biomaterials intensively. Based on the identified publication trends, biomaterials have been playing an irreplaceable role in not only drug delivery systems but also pharmaceutical ingredients for *H. pylori* therapy. Therefore, this section will summarize the current state of development and potential opportunities and challenges in this field, as well as evaluate the main research topics and emerging trends with a critical perspective.

### 2.1. Search methodology

**Figure 1:**
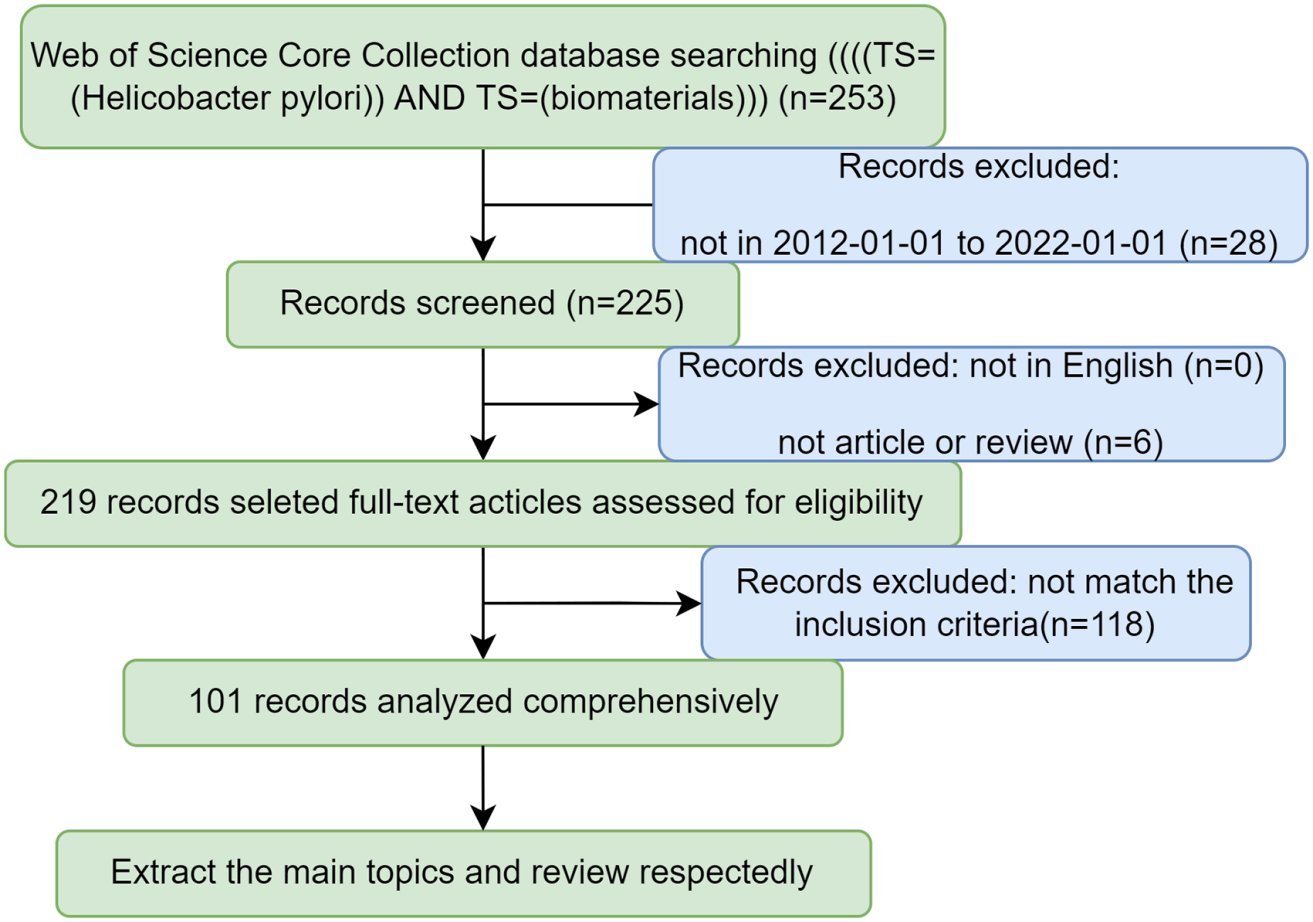
Flow charts of search strategies and filtering methods.

The data used to perform bibliometric analysis in this paper were extracted from the Web of Science Collection Core of Nanchang University Library,^1^ which is an important database platform for domestic and international scholars to retrieve and obtain information about relevant academic literature. We chose to obtain data from the core collection because it owns a stringent evaluation of publications, thus ensuring the high quality of the literature (Zhang et al., 2022). Additionally, the WoSCC database is constantly and dynamically updated and provides the most impactful, relevant, and reliable information (Palechor-Trochez et al., 2021). The search strategy was set as “(TS = (*Helicobacter pylori*)) AND TS = (biomaterials).” Listed as follows are the selection criteria: (Reshetnyak and Reshetnyak, 2017) timespan: ranging from 2012-01-01 to 2022-01-01; (Rajinikanth et al., 2007) type: article or review, language: English. Initially, a total of 219 articles were retrieved. Taking into account the deviations from daily updates to the database, all the data was collected at the same time on April 24, 2022. Two collaborators independently screened the title and abstract of each result excluding irrelevant literature. Ultimately, a total of 101 pieces of literature on the topic of biomaterial therapy for *H. pylori* were collected and downloaded as pure text with full citation and recorded in meta data named biomaterials eradicate *H. pylori*. Subsequently, VOSviewer (version 1.6.18.0), CiteSpace (version 6.1.R1), and R (version 4.2.1) were implemented for further data processing and visual analysis (Chen et al., 2020; Figure 1).

### 2.2. Annual publications and countries distribution

**Figure 2:**
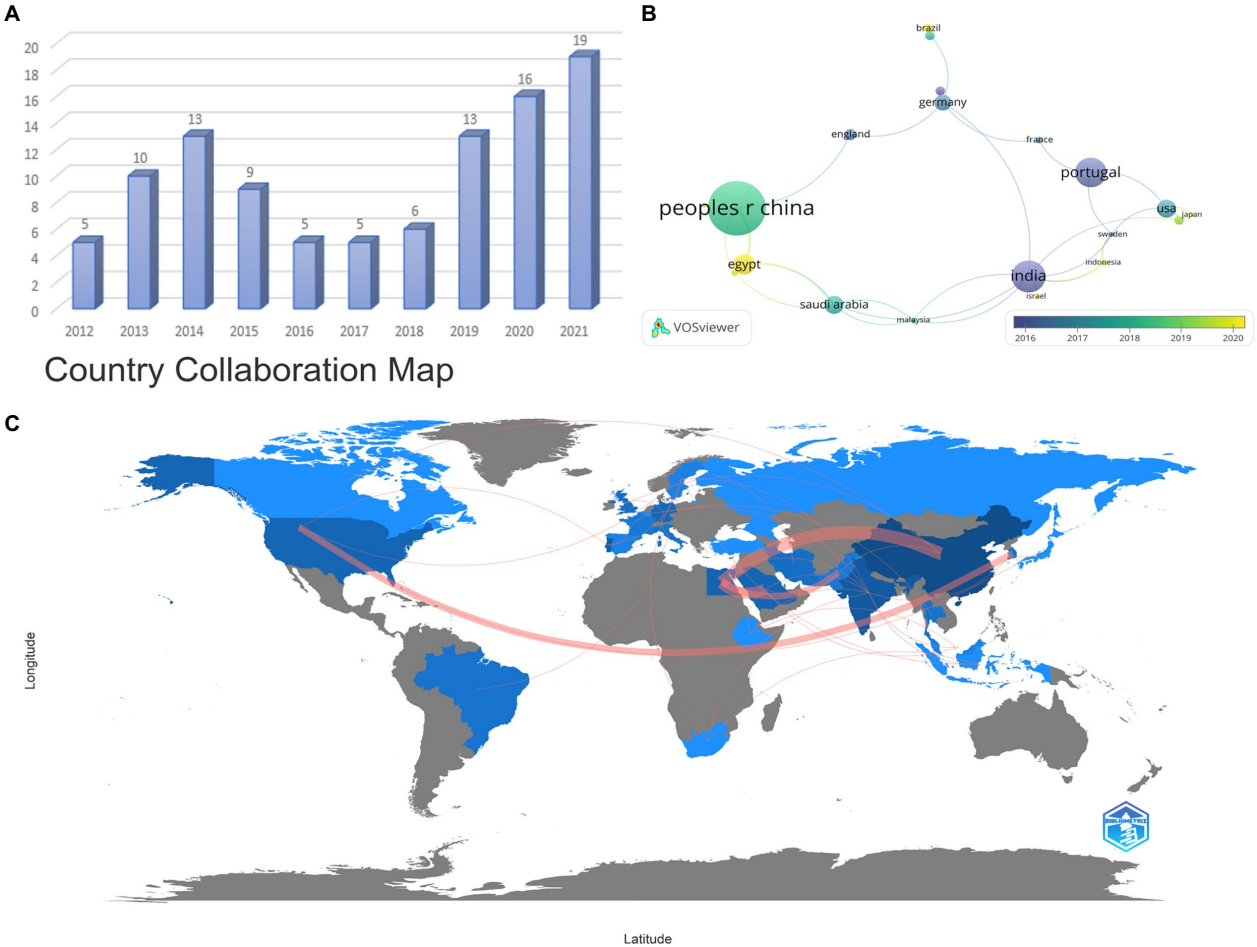
Annual publication status **(A)**, major productive countries distribution **(B)**, and Country Collaboration Map **(C)**. Each node represents a country, and the line between the two nodes indicates that they have a co-occurrence relationship. The larger the node means the greater the volume of national publications. The closer the distance, the stronger the relationship. Moreover, the more yellow the color tends to be, the more cutting-edge the research is **(A,B)**. The red path signifies the partnership between the countries, and the wider the red means the closer the cooperation **(C)**.

The annual distribution of the number of articles published in the past decade is presented in Figure 2A, indicating that the number of articles varies in an S-shape with the year. Although there was a transient declining volume in the intervening years, the trend remains steady increment over the last 5 years. Noteworthily, the volume of publications in the last 3 years is confronted with the most rapid growth, dramatically accounting for more than half of the total. There are adequate reasons to believe that the heat of this field will keep rising sequentially for years to come. As illustrated in Figure 2B, among all countries, China (24 articles) possesses the largest number of published articles compared with other countries. Moreover, India (14 articles) and Portugal (13 articles) present an exceptional contribution in this field as well, respectively ranking second and third. Among all high-producing countries, China and Egypt, respectively, are more strongly engaged with other countries (Figures 2B,C). These discrepancies may be closely related to the local infection situation and the level of research. This division of relationships is beneficial to contribute to scientists exploring where they should establish some important data for those partnerships.

### 2.3. Journal distribution and co-citation analysis

**Figure 3:**
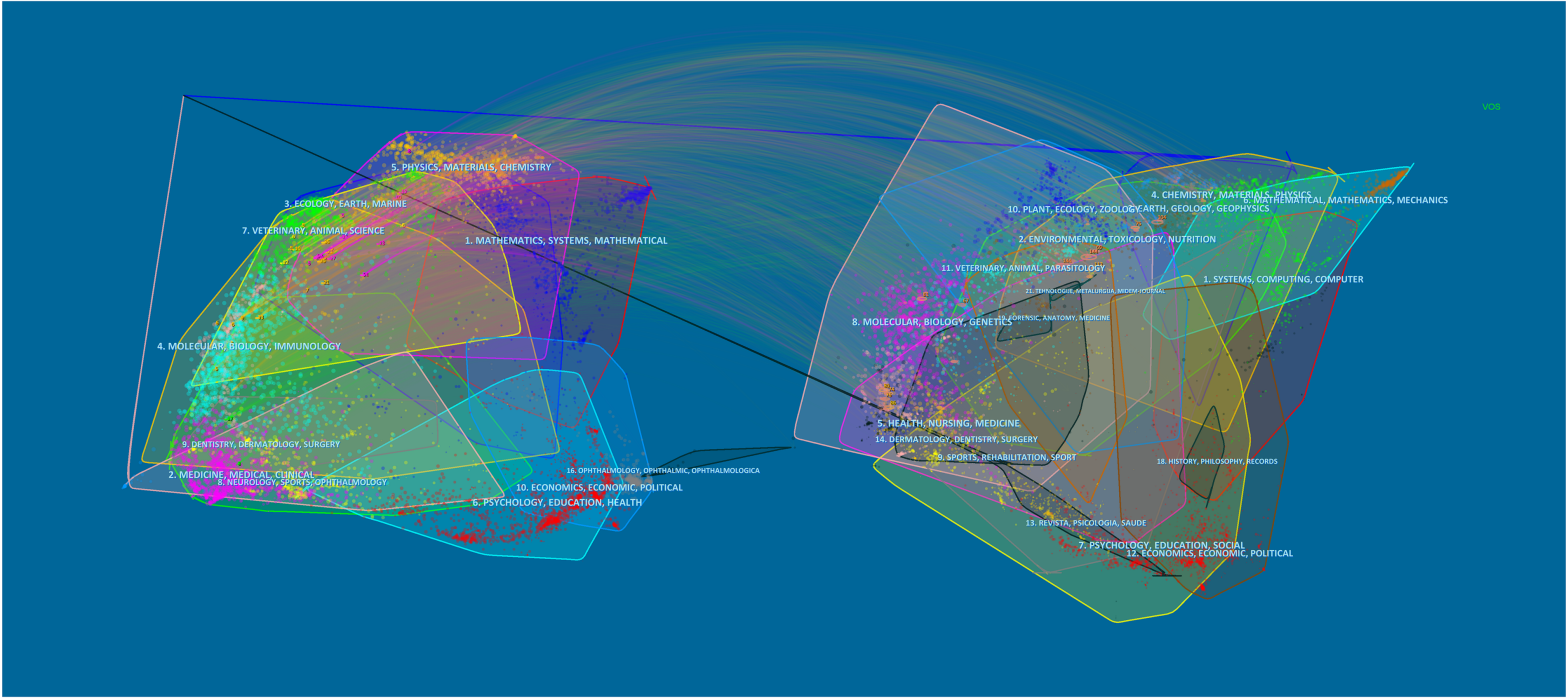
The dual-map overlay of journals on biomaterials eradicating *Helicobacter pylori*. The double map overlay of journals shows the relationship between the two and the distribution among journals, with the citing journals on the left and the cited journals on the right.

Listed below are the journals that published the most papers in the last decade (Table 1). The “International journal of pharmaceutics” (8 articles) owns the highest outputs, followed by “Acta biomaterialia” (6 articles) and “International Journal of biological macromolecules” (6 articles). At the same time, the “International journal of pharmaceutics” is cited most among all the journals, totally reaching 225 times. However, the “Journal of controlled release” possesses the maximum average citations, which demonstrates it is relatively more widely recognized and authoritative. Overall, the top 10 journals with up to two-fifths of the total number of publications have an average impact factor (IF) of 8.0775, among which “Biomaterials” ranks highest (IF = 15.304). Additionally, the double overlay of journals reveals the distribution of relationships between journals. In Figure 3, the left side represents the distribution of the citing literature by journal, reflecting the dominant disciplines to which Science Mapping belongs; the right side is the distribution of the corresponding cited literature by journal, indicating which disciplines Science Mapping primarily cites. The orange and purple paths in the graph illustrate that articles published in the MOLECULAR/BIOLOGY/GENETICS and CHEMISTRY/MATERIALS/PHYSICS directions are frequently cited by articles in the MOLECULAR /BIOLOGY/ IMMUNOLOGY and. PHYSICS/ MATERIALS/ CHEMISTRY directions. Moreover, magazines in the same direction are clustered in the same color block to show the reference relationship between different fields.

**Table 1:** Top 10 leading journals related to *H. pylori* and Biomaterials research from 2012 to 2021.

Journal title	Records	Citations	Average citation	IF (2022)
International journal of pharmaceutics	8	225	28.13	6.510
Acta biomaterialia	6	87	14.50	10.633
International journal of biological macromolecules	6	78	13.00	8.025
Scientific reports	4	66	16.50	4.996
European journal of pharmaceutics and biopharmaceutics	4	42	10.50	5.589
Molecular pharmaceutics	4	100	25.00	5.364
Expert review of anti-infective therapy	3	39	13.00	5.854
Journal of controlled release	3	142	47.33	11.467
Biomaterials	3	134	44.67	15.304
International journal of nanomedicine	3	54	18.00	7.033

### 2.4. The most productive institutions and authors

**Figure 4:**
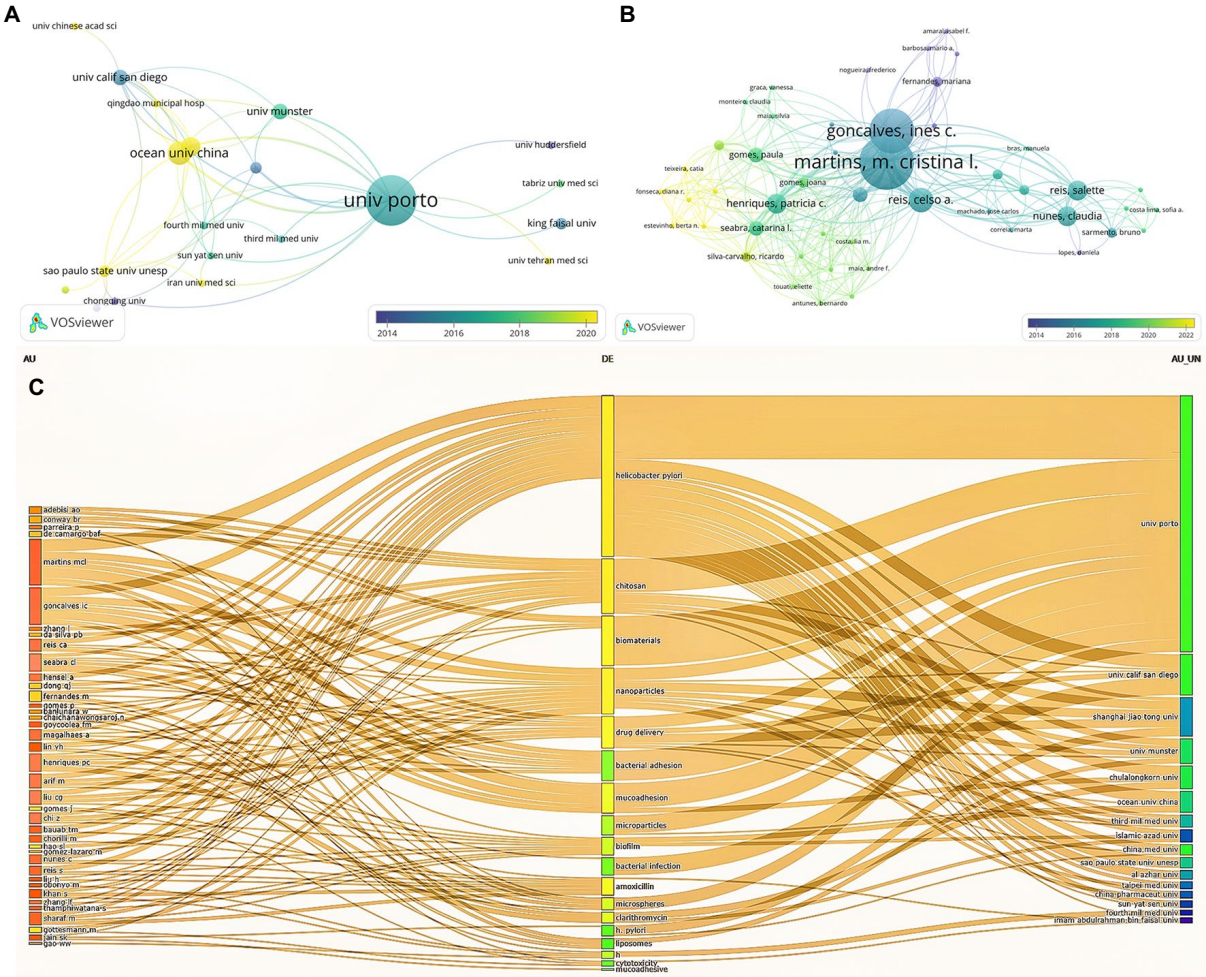
Cited institution **(A)**, author cooperation analysis **(B)**, and Three-Field Plot of author, keyword, and institution **(C)**. Each node represents an institution or an author, and the line between the two nodes indicates that they have a co-occurrence relationship. Moreover, the closer the distance, the stronger the relationship **(A,B)**. In **(C)**, the leftmost square represents the author, the middle is the keyword, and the rightmost is the institution and longer squares mean more research.

Figure 4 illustrates the cluster network of institutions and authors cited. A total of 168 institutions and 540 authors were analyzed, and we selected the top representative results for visualization. Subsequently, we analyzed the total number of publications, citations, and citations per article for the 10 most productive institutions and authors. As exhibited in Figure 4A, the University of Porto (13 articles) has the largest number of publications, more than twice as numerous as the second university. And it is most frequently cited by other institutions, reflecting the high credibility of this institution in the field of biomaterials treating *H. pylori*. Nevertheless, the output of the Ocean University of China has been cited more extensively in recent years, probably owing to its more cutting-edge research direction. Despite the lower volume of publications, the University of California San Diego holds the most citations and average citations, with an average of higher than 100 citations per article, which reveals the relatively advanced quality of this institution’s publications (Table 2). In addition, the network map of each author’s publications and citations over the last decade is depicted in Figure 4B. Among the top 10 authors, each contributing no less than 3 papers, Martins, M. Crastinal is the most prolific contributor to the field. Furthermore, he possesses a total of 253 citations, with the highest citation link strengths (Table 3). A three-Field Plot of authors, keywords, and institutions is exhibited in Figure 4C, which reveals the research orientation of each high-yield author and institution. Figure 4C highlights that the majority of scholars and research institutions have investigated biomaterials for the treatment of *H. pylori* focusing on the areas of chitosan, nanomaterials, drug delivery, and bacterial adhesion. The University of Porto has the broadest research area of any institution, while with a focus on chitosan materials.

**Table 2:** Analysis of the output and citations of the top 10 institutions.

Institute	Records	Citations	Average citations	Link strength
University of Porto	13	279	21.46	57
Ocean University of China	6	39	6.50	26
Al-Azhar University	5	13	2.60	24
University of California San Diego	4	431	107.75	21
University of Münster	4	129	32.25	10
China Medical University	3	151	50.33	14
King Faisal University	3	51	17.00	2
São Paulo State University	3	33	11.00	14
Chongqing University	2	43	21.50	0
China Pharmaceutical University	2	10	5.00	5

**Table 3:** Analysis of the output and co-authorship of the top 10 authors.

Author	Documents	Citations	Average citations	Link strength
Martins, m. Cristina l.	11	253	23.00	46
Goncalves, Ines c.	9	190	21.11	38
Reis, Celso a.	5	82	16.40	28
Arif, Muhammad	5	27	5.40	22
Henriques, Patricia c.	4	49	12.25	20
Sharaf, Mohamed	4	8	2.00	19
Chi, Zhe	4	26	6.50	18
Nunes, Claudia	4	112	28.00	17
Reis, Salette	4	112	28.00	17
Magalhaes, Ana	3	43	14.33	16

### 2.5. The analysis of keywords and frontiers

**Figure 5:**
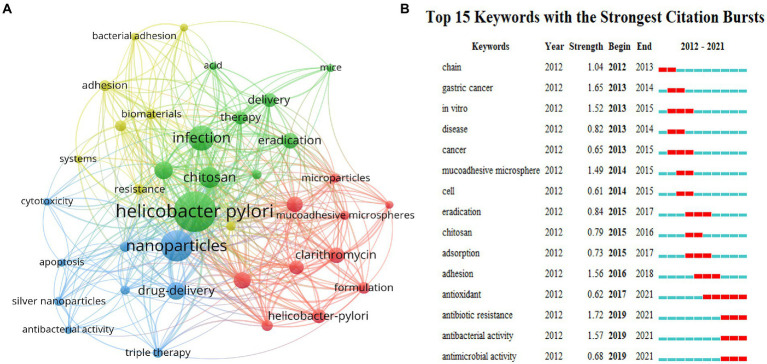
Co-occurrence network of keywords with a minimum of 5 occurrences **(A)**. The top 15 keywords with the strongest citation bursts **(B)**. All the keywords are divided into four clusters, each represented by a different color. Every node symbolizes a keyword, and the line between two keywords indicates that they have a co-occurrence relationship. Additionally, the closer the distance, the stronger the relationship **(A)**. Top 15 bursting keywords in articles related to eradication of *H. pylori* by biomaterials. The blue line represents the time line, and the interval at which bursts were found is indicated by the red portion of the blue timeline, representing the start year, end year, and outbreak duration **(B)**.

As the core of scientific papers, keyword analysis is utilized to track the evolution of knowledge, hot spots, and future research directions. According to Figure 5A, both high occurrences and meaningful keywords of drug or biomaterials are revealed including nanoparticles (Wang et al., 2019), chitosan (Chen et al., 2020), drug-delivery (Darroudi et al., 2021), microspheres (Han et al., 2022), amoxicillin (Butkovich et al., 2021), Clarithromycin (Butkovich et al., 2021), eradication (Butkovich et al., 2021) and release (de Souza et al., 2021). Keywords with a frequency of at least five occurrences were extracted using VOSviewer to obtain a visual network for co-occurrence analysis, and the co-occurrence relationships between various types of keywords were analyzed, resulting in a total of four categories of hotspots for current research. As shown in Figure 5A, all keywords were clustered into four clusters displayed in different colors, and nodes with common attributes were partitioned into a color-coded cluster. Green clusters are mainly associated with *H. pylori* infection, including *Helicobacter pylori*, infection, *in-vitro*, chitosan, treatment, eradication, etc. Blue clusters are mostly relevant to nanoparticles, including nanoparticles, drug delivery, cytotoxicity, apoptosis, etc. Red clusters are largely involved with microparticles and carried drugs regarding amoxicillin, clarithromycin, microspheres and mucoadhesive, etc. Yellow clusters are chiefly concerned with the adhesion and resistance of biomaterials, including adhesion, biomaterials, resistance, etc. The keywords with the strongest bursts in this domain are highlighted in Figure 5B. The red line indicates the time of keyword bursts. Anchored in the burst keywords for discovery, the primary phase features mostly disease and drug keywords concerning gastric cancer, microsomes, and chitosan, suggesting that biomaterials may be applied largely in drug delivery to exert specific functions. In the middle of the period, the keywords “adhesion” and “adsorption” outbreak lasted for 2 years, which illustrates that biomaterials with adsorption and adhesion functions were comparatively promising at that time. Nonetheless, in recent years, with the emergence of drug resistance coming, the development of biomaterials applications has concentrated on antibacterial activity and antibiotic resistance solutions, which will remain a hot topic of research in the future.

### 2.6. Analysis of keyword evolution and continuity

**Figure 6:**
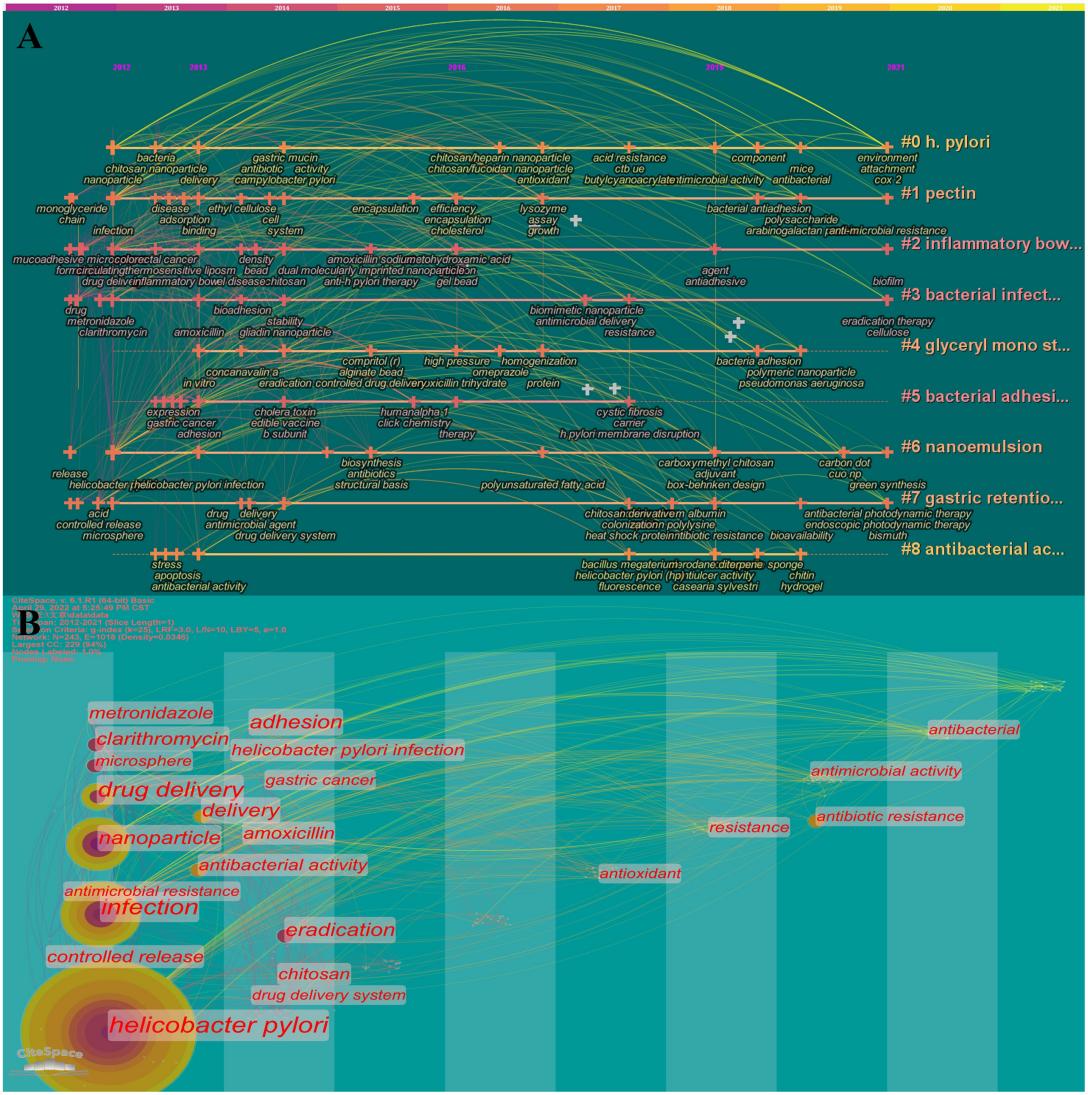
Timeline **(A)** and timezone **(B)** of keywords. 11 clusters are shown in A, and each is labeled with the tag #. The smaller the number, the more keywords are included in the cluster. Each node represents a keyword, and the time when the node appears indicates the time when the keyword emerged. The line between nodes indicates the relationship between keywords and the continuity in time.

The landscape generated using CiteSpace keyword clustering in Figure 6A shows 9 clusters, each labeled with the tag #. The 9 clusters are identified as follows: #0 *H. pylori*, #1 pectin, #2 inflammatory bowel disease, #3 bacterial infection, #4 glyceryl mono stearate, #5 bacterial adhesion, #6 nanoemulsion, #7gastric retention, and #8 antibacterial activity. The evolution of the various materials and methods over time is exhibited in each type of cluster. The close temporal connection between the main keywords is better visualized in Figure 6B, where one vertical bar represents one year. As is reflected that in the first 5 years, miscellaneous biomaterials are predominantly implemented in drug delivery, while the latter 5 years are focused on drug resistance applications. Additionally, emergent keywords are considered indicators of emerging trends. In the following section of “main text,” this study will primarily overview the application of biomaterials in the eradication of *H. pylori*, and then further investigate the facing challenges and potential opportunities.

## 3. Main text

**Figure 7:**
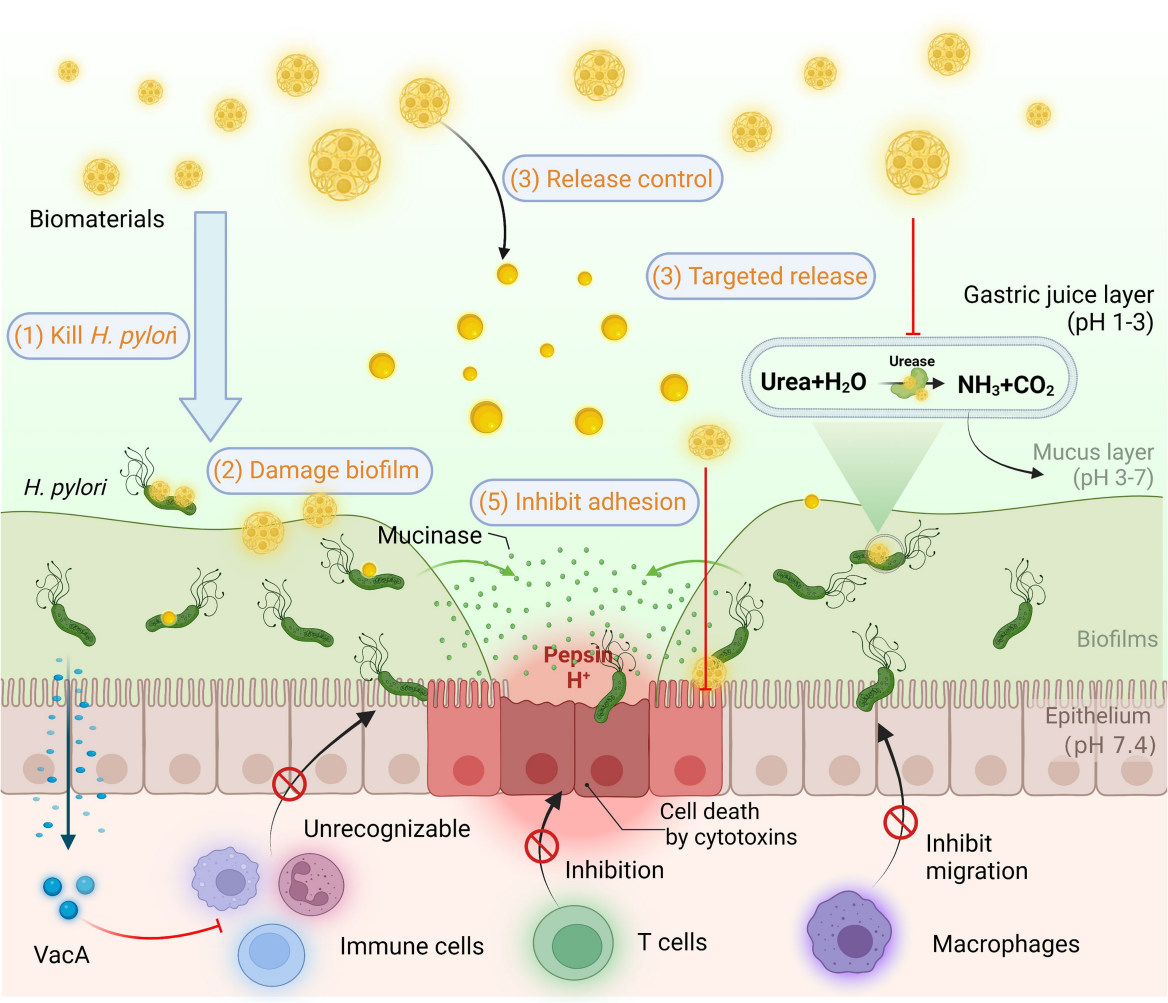
*Helicobacter pylori* infection and common methods of biomaterials in Anti-*H. pylori*. *H. pylori* colonizes and survives for a long time in the body through resistance to acidic environment, biofilm formation, and immune escape. Biomaterials are designed for *H. pylori* eradication by killing *H. pylori*, destroying biofilms, releasing controlled drugs, targeting drug delivery, inhibiting adhesion, and enhancing immunity based on superior biocompatibility.

The difficulty of eradicating *H. pylori* is manifested in multiple aspects. As depicted in Figure 7*, H. pylori* are sheltered from gastric acid by the enzyme urease on the surface of its outer membrane, which breaks down the urea in the surroundings, thus creating a near-neutral microenvironment (Watanabe et al., 2009). Relying on the continuous movement of its flagellum, *H. pylori* penetrated and anchored on the epithelial cell surface of the gastric mucosa, not only effectively avoiding gastric acid erosion, but also significantly minimizing the effect of gastric emptying (Saha et al., 2010). Conversely, most antibacterial drugs are less active or even inactivated in the extremely acidic environment of the stomach (Khan et al., 2022). Even if not catabolized, regular gastric emptying diminishes the concentration of drug accumulation at the site of infection. Since *H. pylori* colonize deep in the mucus layer, the effective contact of antimicrobial drugs with the organism is blocked, making it impractical for the drugs to be efficacious (Vázquez and Villaverde, 2013). Additionally, *H. pylori* successfully evade the host immune response by modifying its outer membrane proteins to escape recognition by the organism, promoting apoptosis of macrophages, inhibiting the migration and uptake of immune cells, suppressing the T-cell immune response, etc. (Kao et al., 2010). Therefore, exploiting biomaterials that reinforce the body’s immune response to *H. pylori* is essential for the eradication of *H. pylori* (Tshibangu-Kabamba and Yamaoka, 2021). Owing to the frequent interchange of DNA, *H. pylori* is susceptible to the development of highly variable strains in continuous infections (Suerbaum et al., 1998). Generally, *H. pylori* infections are persistent, and long-term infections tend to form biofilms, resulting in further resistance (Hathroubi et al., 2018).

The physicochemical properties of biomaterials and their intended routes of delivery have the potential to be systematically tailored to maximize therapeutic efficacy (Khan et al., 2022).

According to our bibliometric analysis, nanoparticles ranked second in the number of occurrences among the occurrence of keywords, trailing only *H. pylori*, indicating nanoparticles play a significant role in the eradication of *H. pylori*. Subsequently, the materials or drugs with a relatively high number of occurrences are “chitosan,” “amoxicillin,” “microspheres” and “clarithromycin” successively. Primarily this is because chitosan particles increase the stability of the nanoparticle structure and ameliorate the drug release rate to some extent. Amoxicillin and clarithromycin are frequently utilized to eradicate *H. pylori* as the primary optional antibacterial antibiotics in triple and quadruple therapies. Noteworthily, keywords including “drug delivery” and “release” are also listed as high-frequency terms, foreshadowing that biomaterials treat *H. pylori* majorly exert effects by improving the release of drug delivery systems. Biomaterials have improved the delivery and efficacy of a range of drug compounds (Langer, 1990). Most of these materials are designed to extend drug retention time and enable further targeted drug delivery, resulting in efficient eradication with reduced dosage and reduced toxicity to the patient.

Given the characteristics and therapeutic limitations of *H. pylori* eradication, the construction of appropriate drug delivery systems for the efficient delivery of existing antimicrobial drugs at the site of infection is a potential platform technology with relatively low risk and high return compared to novel antibacterial drugs (Hussain et al., 2018). On the basis of bibliometric analysis and literature review, we summarize four dominant directions of biomaterials in the field of *H. pylori* eradication from the historical perspective of biomaterials drug delivery research: (Reshetnyak and Reshetnyak, 2017) Release control biomaterials, (Rajinikanth et al., 2007) Targeted biomaterials, (Hooi et al., 2017) Bionic Biomaterials, and (Capurro et al., 2019) Overcoming *H. pylori* drug resistance. We highlight current challenges in the field of drug delivery, breakthroughs in biomaterials research to overcome these barriers, and future considerations and opportunities for biomaterials in clinical applications.

### 3.1. Release control biomaterials

Historically, innumerable clinical practices have demonstrated extremely challenging to eradicate *H. pylori* with single drug therapy (Graham, 2014; Boyanova et al., 2019; Tshibangu-Kabamba and Yamaoka, 2021). The contact time of the antibacterial agent with the organism needs to be sufficiently long. Early reports suggested that by increasing the *in vivo* contact time of the drug with *H. pylori*, the eradication efficiency would be significantly enhanced. Clinical experience has established that the necessity to evaluate the pharmacodynamics and pharmacokinetics of the agents to guarantee optimal bioavailability and concentration in the gastric mucosal fluid after administration is also an essential factor in the difficulty of *H. pylori* eradication (Graham and Dore, 2016). Variable forms of drug delivery systems by using materials with bioadhesive properties are able to maximize the drug residence time in the stomach (Sachin and Karn, 2021). Researchers have developed a variety of gastric retention and release control systems, ranging from bioadhesive systems, mucosal penetration systems, and floating raft systems to micro-motor systems et al., among which biomaterials have played an irreplaceable role in slowing the release rate and increasing drug concentration as novel delivery systems.

#### 3.1.1. Biological adhesion materials

Biological adhesion materials are usually hydrophilic gel polymers containing a multitude of hydrogen bonding groups including carboxy and hydroxyl groups (Wang et al., 2019). The most prevalent polymeric materials utilized for biological adhesion of gastric mucus are chitosan and its derivatives, wheat soluble protein, Polyalkylcyanoacrylate, etc. (Qu et al., 2018). Among mucosal adhesion polymers, deacetylated chitosan is intriguing due to its biodegradability, biological adhesion, and ability to enhance the uptake of active macromolecules (Hejazi and Amiji, 2003). The -NH2 group of chitosan and its derivatives is protonated at the acidic pH of gastric juice and establishes electrostatic interactions with negatively charged gastric mucin and bacterial membranes, thereby exhibiting adhesion properties, and consequently has been developed recurrently for gastric applications (Chaves de Souza et al., 2020; Lang et al., 2020). The lipophilic amino acid residues of maltolysin are capable of interacting with biological tissues, while maltolysin nanoparticles are susceptible to aggregation by pH, temperature, and salt, thus achieving their adhesion properties (Arangoa et al., 2000).

In the recent decade, chitosan nanoparticles or biologically modified materials have been gradually found to be combined with loaded drugs to prolong drug delivery through bioadhesion (Gonçalves et al., 2014). The reacetylated chitosan microspheres developed and optimized by Portero et al. (2002) exhibited controlled water solubility and gelation at acidic pH, leading to prolonged release of encapsulated anti-*H. pylori* drugs. It was revealed that the time of reacetylation is a major factor affecting the drug release and the encapsulation efficiency and antimicrobial activity of the encapsulated compounds. These similar experiments provide a certain foundation for future design optimization of chitosan biomaterials. The *in vivo* and *in vitro* experiments of chitosan nanoparticles against *H. pylori* designed by Luo’s team demonstrated that the anti-*H. pylori* effect of chitosan (CS) nanoparticles solution was negatively correlated with pH when pH was 4–6. Moreover, this work revealed that the anti-*H. pylori* effect of 88.5% deacetylated (DD88.5) CS NPs and 95% deacetylated (DD95) CS NPs was 55 and 75%, respectively. A more in-depth study was conducted by Chang et al. (2020) They observed that at pH 2.0, 4,000 g/ml DD95 suppressed the urease activity of *H. pylori* by 37.86 to 46.53%. In the presence of 50 g/mL of the antibiotics amoxicillin, tetracycline, or metronidazole at pH 6.0 and pH 2.0, *H. pylori* counts decreased by 1.51–3.19 and 1.47–2.82 Log CFU/mL, respectively, while the addition of the same dose and concentration of DD95 under the same conditions strongly depressed the total *H. pylori* counts by 3.67–7.61 and 6.61–6.70 Log CFU/mL. With the loading of antibiotics such as tetracycline and metronidazole, the delivery system suppressed the adhesion of *H. pylori* to cells, thereby promoting the eradication rate of *H. pylori*. Therefore, biological adhesion materials are promising carriers for the controlled delivery of antimicrobial agents to the gastric cavity and therefore for the eradication of *H. pylori*, a pathogen closely associated with gastric ulcers and possibly gastric cancer.

#### 3.1.2. Mucus-penetrating system

Given that *H. pylori* are colonized under the mucus layer, mucus-penetrating agents facilitate drug delivery to the site of infection and thus enhance eradication rates (Chmiela and Kupcinskas, 2019). Depending on the properties of mucin, a major component of mucus, it is assumed that nanoparticles with hydrophilic, negatively charged surfaces and small particle sizes are capable of effectively penetrating the mucus layer (Nogueira et al., 2013). Previous research has established that positively charged chitosan nanoparticles facilitate mucosal penetration. However, Zhang et al. (2018) designed a biomaterial in which nanoparticles were electrostatically self-assembled with antigen and cell-penetrating peptide (CPP) and then coated with a “mucus-inert” PEG derivative that gradually dissociated from the nanoparticles in the mucus, exposing the CPP-rich core and thus enabling penetration. The experiment results demonstrated that the nanoparticles overcome the mucus barrier for active drug delivery after oral administration. Compared with the positively charged chitosan nanoparticles, the PEG-modified nanoparticles weakened the interaction with mucin and could effectively penetrate the mucus layer to reach the infection site, which further strengthened the elimination rate of *H. pylori*. Consequently, the whole material reduces the contact with gastric mucin and also achieves the effect of drug delivery by osmosis.

In addition to the construction of nanoparticles with a hydrophilic surface, negative charge, and small particle size, the applied magnetic field is capable of facilitating the effective penetration of the drug delivery system into the gastric mucus layer and reaching the site of *H. pylori* infection (Silva et al., 2009). For instance, Chitosan/polyacrylic acid particles co-loaded with superparamagnetic iron oxide nanoparticles and amoxicillin prepared by Yang et al. (2020) were employed as drug nanocarriers for *H. pylori* eradication therapy. The nanocarriers noticeably enhanced the penetration into the gastric mucus layer and improved the eradication of *H. pylori* when exposed to an applied magnetic field. The results showed that all the nanoparticles accumulated at the bottom of the mucus layer after 10 min of the applied magnetic field, indicating that the mucus penetration efficiency of the prepared magnetic nanoparticles could be controlled by the applied magnetic field. Similarly, Walker et al. (2015) prepared a magnetic microhelix system with immobilized urease on the surface by simulating the movement of *H. pylori* through the gastric mucus layer. The results exhibited that the applied magnetic field allowed the system to advance efficiently in the gastric mucus layer, while the surface-immobilized urease significantly promoted the mobility of the microparticles. Therefore, these nanocarriers prolong the residence time of the drug in the stomach, reducing the drug dose and treatment time required for *H. pylori* eradication therapy.

#### 3.1.3. Floating raft system (FRS)

Among the dwelling drug delivery systems, the floating raft system achieves drug gastric retention by floating on the gastric contents for prolonged drug delivery as a result of its low density and has been evaluated for maintaining drug delivery and targeting (Thombre and Gide, 2016). Conway’s group (Adebisi et al., 2015) developed calcium alginate microspheres by ionic gelation and modified them with chitosan and oil to optimize float ability, adhesion, and drug release. The experimental results revealed that the floating beads remained for at least 24 h. More than 75% of the beads were adherent to the gastric mucosa for more than 8 h and guaranteed drug release, indicating the fresh dosage form ensures better retention time in the stomach than the convention. Additionally, Rajinikanth et al. (2007) demonstrated the feasibility of prolonging the gastric residence time and release rate of metronidazole utilizing an FRS prepared from ion-sensitive *in situ* gels. FRS consists of sodium alginate and gellan gum, sodium citrate and calcium carbonate, and lipids. Release kinetic studies of the selected formulation revealed that FRS had a short-term gelation lag time (3 s) and a duration of up to 24 h, with a reliable slow release of the drug. The refined properties of the selected FRS make it an excellent candidate for gastric-targeted eradication of *H. pylori*.

#### 3.1.4. Nanomotor system

The protracted administration of PPI is prone to side effects involving osteoporosis, vitamin C deficiency, etc. (Pouwels et al., 2011; Heidelbaugh, 2013). Nevertheless, bio-inspired design principles and advances in nanomaterials have generated significant advances in the field of intra-gastrointestinal drug delivery, especially in nano/micro motors, which are essentially chemically neutralized to modulate the harsh acidic environment to neutral and avoid reducing the efficacy of the drug. Micromotors commonly refer to chemically driven nanomotors, which are small devices facilitated by catalytic reactions in liquids (Sánchez et al., 2015). Artificial micromotors enable self-propulsion in the stomach, enhanced retention of intestinal fluid in the gastric mucosal layer, and targeted delivery in the gastrointestinal tract. Walker et al. (2015) demonstrated the ability of magnetic micro propellers to move through gastric mucus gel by simulating the mucus permeation strategy of *H. pylori*.

In regard to eradicating *H. pylori*, Wu et al. (2021) report a nanomotor that allows small molecules of clarithromycin, calcium peroxide nanoparticles (CaO_2_) and platinum nanoparticles to be loaded into the motor *via* ultrasound. The nanomotor can rapidly consume gastric acid and temporarily neutralize gastric acid by the chemical reaction of CaO_2_. The reaction of CaO2 with gastric juice has been demonstrated by *in vivo* experiments to result in rapid consumption of protons, thereby temporarily neutralizing acid without affecting normal gastric function. In particular, the acid-driven nanomotors can be effectively loaded with antimicrobial drugs and exhibit prominent bactericidal activity. Similarly, Wang et al. (de Ávila et al. 2017) experimented with the efficient propulsion of magnesium-based micromotors in an acidic gastric environment. Upon temporary depletion of gastric acid, they were actively and persistently retained in the gastric mucosa. The experimental results illustrate that acid-driven magnesium-based micromotors efficiently load clinical doses of drugs and exert significant *H. pylori* eradication capabilities. These conclusions implicate the nanomotor as a promising alternative to PPI in *H. pylori* eradication.

#### 3.1.5. Magnetic release control biomaterials

Magnetic drug delivery particle carriers are a tremendously effective modality for delivering drugs to localized disease sites in the gastrointestinal tract. The speed of passage through the GI tract can be slowed down at specific locations by external magnets, thus altering the time and extent of drug absorption in the stomach or intestines (Häfeli, 2004). Furthermore, Thamphiwatana et al. (2013) attached chitosan-modified gold nanoparticles to the outer surface of doxycycline-loaded anionic liposomes. Under a gastric acidic environment, the gold nanoparticles spontaneously bound to the surface of the anionic liposomes by the mutual attraction of heterogeneous charges, which effectively delayed the drug release. Once the neutral pH environment was reached, the surface charge of gold nanoparticles was reduced to detach from the liposomes, exposing the drug-loaded liposomes, which released the drug by fusing with *H. pylori* cell membranes. Compared with free doxycycline, the gold nanoparticle-encapsulated liposomes displayed a stronger antibacterial effect against *H. pylori*.

With multidisciplinary cross-fertilization, Yang et al. (2014) Chitosan/polyacrylic acid particles physically co-loaded with superparamagnetic iron oxide nanoparticles and amoxicillin (SPIO/AMO@PAA/CHI) were used as drug nanocarriers for *H. pylori* eradication therapy. *In vitro* and *in vivo* results showed that the designed SPIO/AMO@PAA/CHI nanoparticles were biocompatible and could retain the biofilm inhibitory and bactericidal effects of amoxicillin against *H. pylori*. In addition, the mucosal adhesion properties of chitosan allow SPIO/AMO/PAA/CHI nanoparticles to adhere to the gastric mucus layer and to rapidly cross the mucus layer after exposure to a magnetic field. Consequently, the application of this nanocarrier allows for prolonged drug residence time in the stomach, reduced drug doses, and treatment cycles for *H. pylori* eradication therapy (Yun et al., 2015).

### 3.2. Targeted biomaterials

To date, polymers that respond to numerous different triggers have been developed and explored for biomaterial applications (Chen et al., 2019). The aims of each of these systems are to promote drug accuracy, as well as to augment the quality of life of patients. Recently, stimulus-responsive “smart” biomaterials have been designed to initiate drug release in response to a range of environmental stimuli (e.g., pH, urel, photo response). In this section, we highlight specifically targeted drug delivery biomaterials for the treatment of *H. pylori* from multiple perspectives.

#### 3.2.1. PH-response biomaterials

pH-sensitive specific materials hold promising prospects for a widespread application in anti-*H. pylori* drug delivery systems. Su et al. (2016) synthesized a poly (glutamic acid-arginine) complex peptide, which exhibited different morphologies in different pH environments to control drug release. At pH 2.5, the nanoparticles formed by peptide self-assembly were dense and intact spheres with little release of amoxicillin. The peptide nanoparticles exhibited a diffuse state when pH 7.0, therefore contributing to the steady release of amoxicillin. Furthermore, Jing et al. (2016) designed and synthesized a pH-response drug delivery system against *H. pylori* using UCCs/TPP nanoparticles encapsulated with amoxicillin. The results showed that the amoxicillin- UCCs /TPP nanoparticles had superior PH-sensitivity and could delay the release of amoxicillin in gastric acid, enabling the effective delivery and targeting of the drug to the survival region of *H. pylori*. The protective effect of these bio-nanoparticles on amoxicillin and the controlled release resulted in the inhibition of *H. pylori* growth about 5.1 times higher than that of single amoxicillin.

In a further breakthrough, low molecular weight rockrose gums/CS-N-Arg NPs have been developed (Lin et al., 2017). The NPs were further cross-linked with genipin to obtain pH-responsive nanogels. Ultimately, they were found to exert inhibitory effects on *H. pylori* adherence and preventive effects on pathogen-induced gastric epithelial cytotoxicity. Recently, Yan et al. (2021) report a pH-responsive persistent luminescence enzyme for *in vivo* imaging and inactivation of *H. pylori*. The persistent luminescence enzyme, composed of mesoporous silica-coated sustained luminescence nanoparticles, Au nanoparticles, and chitosan-benzoic acid, exhibits good resistance to gastric acid corrosion and exhibiting pH-activated dual-nano activity, thereby catalyzing the performance of bactericidal reactive oxygen species.

#### 3.2.2. Urel targeted materials

The urea transport channel protein (Urel) is one of the most essential factors for the survival of *H. pylori* in the stomach, as it modulates the opening and closing state according to the pH value of the stomach (Cui et al., 2019). UreI is utilized as a target for delivering drugs to block the transport of urea and disrupt the survival environment of *H. pylori* so as to make it fail to colonize the gastric mucosa, thereby achieving the eradication of *H. pylori*. Building on the UreI-mediated targeted drug delivery system, scientists have invented biological nanomaterials for the specific eradication of *H. pylori*. Luo et al. (2018) reported that ureido-conjugated chitosan showed the ability to target UreI specifically expressed by *H. pylori*. The ability of the drug delivery system constructed on the basis to eliminate *H. pylori* was significantly enhanced. Analogously, Cong et al. (2019) coupled carboxymethyl chitosan modified with stearic acid to urea and presented exceptional *H. pylori* targeting and anti-*H. pylori* efficacy as well.

#### 3.2.3. Photo responsive biomaterials

Photo-responsive therapy, a therapeutic technique in which a photosensitizer oxidizes biomolecules and causes irreversible damage by generating reactive oxygen species under laser irradiation, has attracted increasing attention as a promising strategy for eliminating bacteria (Huang et al., 2012; Jeong et al., 2014). To develop a photo-responsive *H. pylori*-based therapeutic regimen, Na et al. (Im et al., 2021) proposed a photo-responsive system targeting *H. pylori* consisting of multiple 3′-sialoyl lactose (3SL)-coupled poly (l-lysine)-based photosensitizers (p3SLP). P3SLP achieves specific delivery of *H. pylori*-based drugs through the specificity between 3SL and sialic acid-binding adhesin (SabA) on the membrane of *H. pylori* interaction to achieve specific *H. pylori*-based drug delivery (Garcez et al., 2010). This is principally attributed to the fact that one of the outer membrane proteins of *H. pylori* is sialic acid-binding adhesin (SabA), while the 3SL receptor is not expressed in mammalian cells thus avoiding undesirable phototoxicity to normal cells (Ling et al., 2012). The authors’ gastrointestinal assays in *H. pylori*-infected mice exhibited that the photo-responsive system had a pronounced *H. pylori*-specific antibacterial effect with no side effects on normal tissues. Additionally, an anti-inflammatory response was observed at the site of infection following p3SLP treatment. Although the clinical application of photo-responsive treatment of *H. pylori* is still an underdeveloped field, this approach does not contribute to adverse drug resistance compared to conventional antibiotic-based treatment (Demidova and Hamblin, 2004; Dai et al., 2009). However, the specific wavelength of laser light required for a particular type of photosensitizer varies from one to another. Therefore, we can continuously explore more photosensitizers to improve the potential of photosensitization therapy for *H. pylori* eradication (Park et al., 2016).

### 3.3. Bionic biomaterials

Cell membranes have attracted extensive attention in the field of biomedicine in recent years due to their properties concerning prolonged circulation time *in vivo* and homologous targeting. For example, natural cell membranes are encapsulated with nanoparticles in their cores as a shell, allowing the nanoparticles to possess the biological properties of natural cells.

#### 3.3.1. Liposomes

Liposomes (LPs) LPs are defined as lipid vesicles composed of one or more phospholipid bilayers, with spherical shapes and sizes between 25 and 1,000 nm. They can encapsulate lipophilic and hydrophilic drugs in lipid membranes and aqueous cores, respectively. LPs show many advantages, such as the flexibility to change their chemical composition and, moreover, allow for surface functionalization or targeted delivery. Considering the cell membrane-like structure of LPs, they exhibit good biocompatibility, low toxicity, etc. Thamphiwatana et al. (2013) evaluated the activity of LPs containing integrated linolenic acid (LLA), naming the system LipoLLA, against *H. pylori*. Several free fatty acids, including LLA, have been investigated as new drugs because of their antibacterial activity against various bacteria. In this study, fusion with bacteria was confirmed by a lipophilic fluorophore label, illustrating that LipoLLA was able to cause some damage to the bacterial membrane.

Recently, Martins’ team employed precrol®ATO5 and Miglyol®812 as lipids and Tween®60 as a surfactant to prepare nanostructured lipid carriers (NLC). Seabra et al. (2018) demonstrated that NLC, even without any drug loading, is capable of destroying *H. pylori* at low concentrations. NLC is designed to rapidly bind and destroy the *H. pylori* bacterial film without affecting other bacteria, resulting in bacterial death. This study reveals that NLC is a bright avenue to explore in the quest for innovative antibiotic-free treatments against *H. pylori* infection.

#### 3.3.2. Membrane biomaterials

The application of natural cell membranes in the field of biomimetic nanomedicine has attracted much attention in recent years on account of their prolonged circulation time *in vivo* and outstanding biocompatibility. For instance, natural cell membranes are encapsulated with nanoparticles in their cores as a shell, thus giving the nanoparticles the biological properties of natural cells. Angsantikul et al. (2018) coated gastric epithelial cell membranes with clarithromycin-loaded polymers, and the nanoparticles preferentially adhered to the surface of *H. pylori* and presented better therapeutic effects in an *in vitro* test. In addition to host cell membrane mimicry, pathogenic cell membrane mimicry nanoparticles could interfere with the interaction between pathogenic bacteria and the host. The NPs compete with *H. pylori* for the binding sites on the host cells and detach the adherent *H. pylori*, exerting a noteworthy anti-adhesive effect (Zhang et al., 2019). These explorations are proved to be a pioneering choice as a coating material to boost the biocompatibility of drugs, exhibiting properties concerning immune escape, high circulation time, moderating elimination of the reticuloendothelial system, mimics cellular glycocalyx to prevent serum protein adsorption and counteract complement response.

#### 3.3.3. Phage biomaterials

Specially modified phages are available to bind to specific pathogenic bacteria. Aiming to strengthen the antibacterial ability of phages, genetic engineering and chemotherapeutic drug coupling technologies have been established for the modification of phages and drug delivery. Cao et al. (2000) constructed a modified phage M13 to express a shell protein that fused with *H. pylori* cell membrane surface-specific antigen. The results indicated that the recombinant phage M13 exhibited bactericidal effects and specifically inhibited the growth of six *H. pylori* strains. Moreover, oral pretreatment with M13 significantly attenuated the colonization of *H. pylori* in the stomach of mice. Sequentially, Ardekani et al. (2013) successfully exploited an M13 phage-based nanovirus. Through sodium dodecyl sulfate-polyacrylamide gel electrophoresis and Western blotting analysis, the nanovirus was confirmed to inhibit urease activity, further disrupting the survival environment of *H. pylori*. There are few studies on phages against *H. pylori*, and no studies have been conducted on their application as drug carriers in the field of anti-*H. pylori*. As the mechanism of phage bactericidal activity is completely different from that of antibacterial drugs, phage therapy is expected to be an attractive approach to addressing the multidrug resistance of *H. pylori*. However several human gut microbiota research studies have demonstrated that phages perform a function in intestinal homeostasis (Ferreira et al., 2022). Currently, phages are thought to precisely affect the intestinal microbiota and exert beneficial effects on numerous gastrointestinal disorders (Muñoz et al., 2020). However, whether the M13 phages discussed above have a specific impact on the intestinal microbiota still deserves a lot of investigation.

### 3.4. Overcoming *Helicobacter pylori* drug resistance

**Figure 8:**
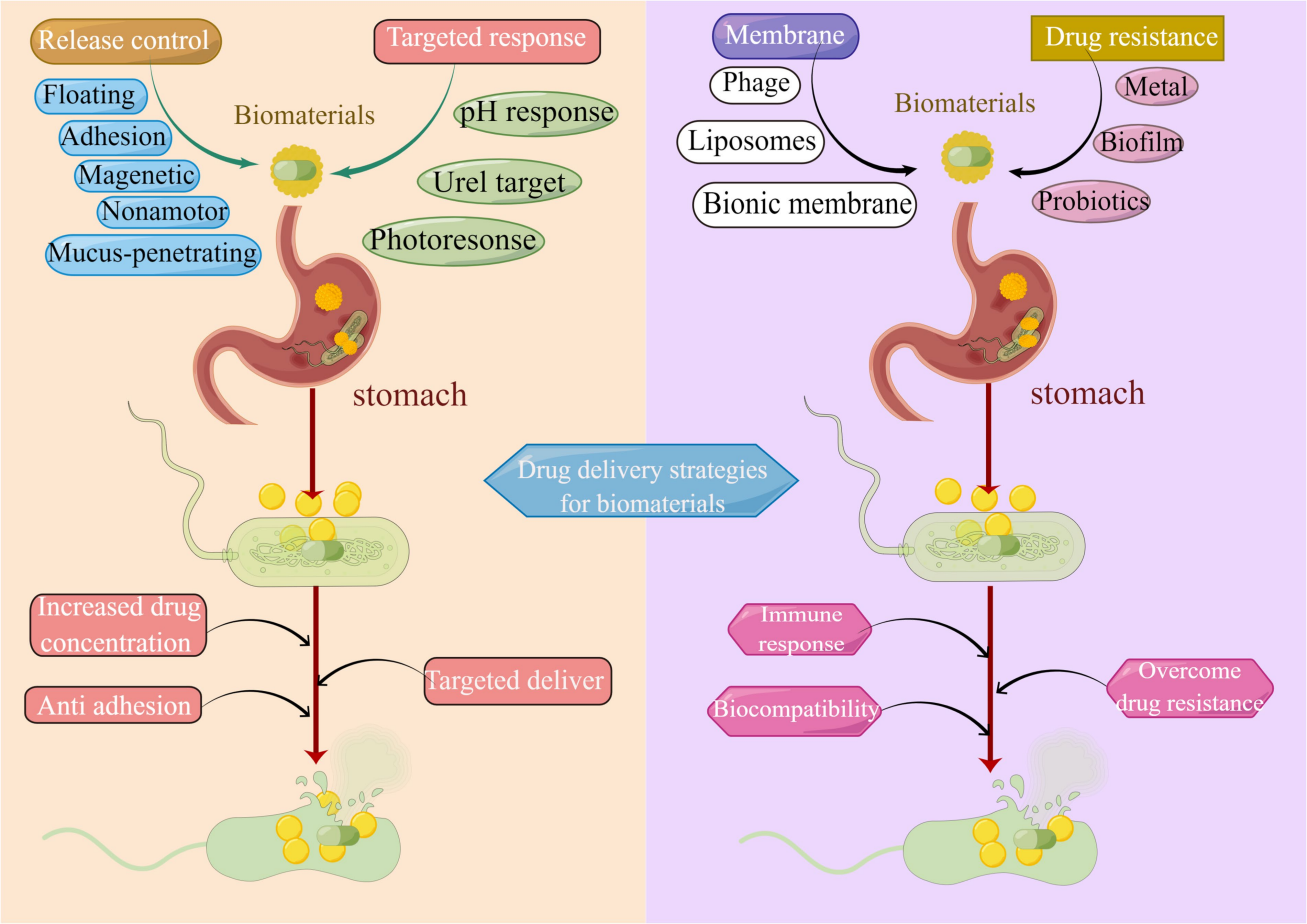
Drug delivery strategies of biomaterials in *H. pylori* eradication. Common strategies utilized for drug release control include bioadhesion, mucosal penetration, floating raft, nanomotor, and magnetic materials. Methods applied for targeted drug delivery are pH response, Urel targeting, and photo response. Biomimetic biological membrane materials are liposomes, cell membranes, and phages. Strategies to overcome drug resistance include metal materials, probiotic materials, and the elimination of biofilm.

Some researchers have recognized *H. pylori* gene mutations, for instance, infB and rpl22 (Binh et al., 2014), as the root cause of drug resistance (Gong and Yuan, 2018). Currently, the majority of clinical *H. pylori* therapies are antibiotic therapies. Each antibiotic is associated with a specific target, and when the corresponding target is structurally altered, the antibiotic is prevented from exerting its original efficacy (Gong and Yuan, 2018). The integration of a multi-target antibacterial mechanism into the drug delivery system is expected to reduce the drug resistance of *H. pylori*. As portrayed in Figure 8, multiple target eradication modalities for *H. pylori* have been developed in recent years. Metallic materials in disrupting *H. pylori* biofilm and urease activity (de Reuse et al., 2013), and probiotic materials in relieving inflammation, mitigating *H. pylori* adhesion, and enhancing immune response (Zhang et al., 2022) have all been demonstrated to be promising alternative therapeutic modalities to overcome antibiotic resistance.

#### 3.4.1. Metallic biomaterials

Metal nanoparticles exert antibacterial effects through metal ion release, oxidative stress, and non-oxidative stress (Zaidi et al., 2017). As a result of various antibacterial mechanisms, metal nanoparticles are efficient at low concentrations and not easily induced to develop drug resistance. It was demonstrated that the antibacterial activity of silver nanoparticles not only inhibited the respiratory system and biofilm formation of *H. pylori* but also directly interfered with the nickel in the urease of *H. pylori* to inactivate the urease, exerting the antibacterial efficacy (Amin et al., 2012). Sreelakshmi et al. (2011) synthesized silver nanoparticles using Glycyrrhiza glabra root extract, which has known therapeutic activity in the treatment of gastric ulcers. In the agar diffusion test, the nanoparticles showed activity against *H. pylori* and can be considered a new method to eradicate this bacterium in the treatment of gastric ulcers.

Besides silver, other metals have also been used in the biosynthesis of nanoparticles as an alternative treatment for *H. pylori* infection. It is worth mentioning that ZnO NPs have been approved and generally recognized as safe for normal cells. Chakraborti et al. (2013) employed polyethyleneimine (PEI) functionalized ZnONPs (ZnO-PEI NPs), which greatly reduced the surface energy of ZnO NPs. The ZnO-PEI NPs were effective against *H. pylori* metronidazole-resistant strains, and their mechanism of action included promoting the production of intracellular reactive oxygen species and causing cell membrane and RNA damage. Wu et al. (2019) designed bifunctional magnetic nanoparticles placed in a moderate AC magnetic field to locally deposit heat and effectively inhibit the growth and virulence of *H. pylori in vitro*. The survival rate of *H. pylori* was reduced to 1/7 and 1/5 after treatment with amoxicillin-loaded metal nanoparticles compared with that of amoxicillin alone or blank metal nanoparticles, respectively. The mechanism may be the damage of the cell membrane and increased penetration of amoxicillin into *H. pylori*, and thus elevated the deracinating efficiency of drug-resistant strains. In clinical applications and against intestinal microbial infections, pH-sensitive cis-aconitate anhydride-modified anti-*H. pylori* conjugated gold nanostars synthesized by Zhi et al. (2019). The near-infrared laser photothermal treatment enhanced the bactericidal effect, reduced the emergence of *H. pylori* drug resistance, and even eradicated drug-resistant strains of *H. pylori* isolated from clinical patients. Additionally, most patients were eliminated from the body within 7 days after the completion of treatment. Therefore biomaterials do hold a promising clinical translation in the eradication of *H. pylori*. However, the side effects of metal nanomaterials and their dosages on normal tissues are not well investigated. Kim et al. (2010) evaluated the toxic effects of 30 mg/kg, 125 mg/kg, and 500 mg/kg of silver nanoparticles injected into rats. As a result, rats injected with more than 125 mg/kg of silver nanoparticles exhibited toxic reactions and weight loss in the liver. The team revealed that the minimum dose at which harmful effects were observed and the minimum dose at which adverse effects were observed were determined to be 125 mg/kg and 30 mg/kg, respectively. Accordingly, the tendency of most metal nanoparticles to accumulate in organs such as the kidney (Garcia et al., 2016), liver, lung, and spleen, as well as the under-studied toxicity of metal nanoparticles to gastric cell lines, have limited the use of metal nanoparticles in the treatment of *H. pylori*.

#### 3.4.2. Elimination of biofilm

Bacterial biofilm formation is an overwhelming mechanism of bacterial drug resistance (Chen et al., 2018). Since the discovery of *H. pylori* biofilm in clinical patients, the problem of drug resistance caused by *H. pylori* biofilm has become a hot topic of interest. It has been exhibited that natural products containing N-acetylcysteine, polysaccharide sulfate, and curcumin hold the ability to inhibit the formation of *H. pylori* biofilm, while alginate lyase can eliminate *H. pylori* biofilm by disrupting the biofilm structure (Bugli et al., 2016). On this foundation, Gurunathan et al. (2015) confirmed that the silver nanoparticles stabilized with N-acylhomoserine lactase significantly inhibited the formation of *H. pylori* biofilm, which may be related to the inhibition of biofilm population sensing (Gopalakrishnan et al., 2020).

#### 3.4.3. Hydrogen therapy

Interestingly, hydrogen therapy has previously been applied to eradicate *H. pylori*. Wang’s group has presented a pH-responsive metal–organic backbone hydrogen nanoparticle (Pd(H) @ZIF-8). The nanoparticle was wrapped in ascorbyl palmitate hydrogel to target and adhere to the site of inflammation by electrostatic interactions, and thereafter hydrolyzed at the site of inflammation by an enriched matrix metalloproteinase. The released Pd(H) @ZIF-8 nanoparticles are further broken down by gastric acid to produce zinc ions (Zn^2+^) and hydrogen gas, thus effectively disintegrating *H. pylori* and alleviating inflammation while repairing the damaged gastric mucosa. Unexpectedly, animal experiments have demonstrated that this biomaterial also can avoid intestinal flora dysbiosis, thus providing a more precise, effective, and healthy strategy for the treatment of *H. pylori* infection (Zhang et al., 2022).

#### 3.4.4. Probiotic biomaterials

Probiotics are defined as live microorganisms that, when given in sufficient amounts, provide benefits to the host (Chen et al., 2019). Recent investigations have indicated that probiotics are capable of increasing antibiotic activity and may block some resistance mechanisms. For instance, in a meta-analysis, the addition of probiotics to triple therapy was observed to enhance the eradication rate of *H. pylori* by >12% (Lau et al., 2016). Furthermore, probiotics dramatically minimize the adverse effects of treatment regimens ranging from maintaining intestinal flora homeostasis, moderating inflammation, and diminishing *H. pylori* adhesion, to elevating the immune response (Lionetti et al., 2010). The antimicrobial, immunomodulatory, and antioxidant properties of lactoferrin increased when it was attached to the surface of bionic nanocrystals (Nocerino et al., 2014). Fulgione et al. (2016) designed a combination material consisting of bionic hydroxyapatite nanoparticles and *Lactobacillus paracasei* probiotic supernatant based on this efficacy as an alternative therapy for *H. pylori* infection. The experimental results demonstrated that the supernatant group of lactoferrin (200–600 μg/mL) plus *Lactobacillus paracasei* had higher antibacterial activity than the conventional antibiotic combination (amoxicillin 200–600 μg/mL, clarithromycin 200–600 μg/mL), even lower levels of pro-inflammatory cytokines such as IFN-γ and higher concentrations of IgG antibodies in the body. This further reveals that probiotics may ameliorate *H. pylori*-induced gastrointestinal inflammation and improve immunity, thus increasing eradication rates (Lin et al., 2020). Consequently, the combination of probiotics and biomaterials is anticipated to be an attractive approach to drug resistance or adjuvant therapy for *H. pylori* infection (Chen et al., 2018).

## 4. Conclusion

In a nutshell, this paper primarily investigates the application of biomaterials in the eradication of *H. pylori* in the last decade using bibliometric analysis from multiple perspectives, ranging from the number of annual publications to hot keywords of research. Subsequently, we explored the research hotspots in each period and conducted a comprehensive literature review with reference to the evolution of the keywords. Moreover, this study analyzed the characteristics of *H. pylori* infection and the underlying reasons for its difficult eradication and focused on drug delivery strategies and novel therapeutic approaches to maximize *H. pylori* eradication rates while mitigating drug resistance.

With the evolution of biomaterials for drug delivery in the last decades, there has been a dramatic expansion in the development of biomaterials for controlled release, using adhesion, floating raft, nanomotor, and magnetic-based mechanisms to control the release rate of the incorporated drug. In these biomaterials, chitosan exerts a constructive role. However, the anti-*H. pylori* activity of chitosan and its derivatives are influenced by various parameters, with significant discrepancies in the degree of deacetylation, modification groups, and molecular weight required for different flora. Therefore, their safety and stability in clinical applications require further refinement and validation. In recent years, targeted “smart” biomaterials have been designed to initiate target responses to *H. pylori* based on a range of environmental stimuli regarding pH, urel, and photo response. Besides, novel biomaterials in other fields have been developed that can be remotely triggered by stimuli including ultrasound, electric current, and magnetic fields for on-demand drug delivery. Hence there is considerable potential for targeted drug delivery against *H. pylori*. In terms of cell membranes, liposomes and biofilm materials have been engineered as novel bionic drug delivery systems owing to their extended *in vivo* circulation time and homologous targeting properties. Furthermore, Phage therapies are emerging in the field of anti-*H. pylori* and their specificity, low resistance, and extensive sources make them a promising alternative for the prevention and control of *H. pylori* infection. Of necessity, phage therapy presents problems in terms of dose and duration of treatment as well as potential toxicity and needs to be researched extensively as a novel drug delivery system. Materials such as metallic biomaterials that perform the function of disrupting the biofilm formed by *H. pylori* have an irreplaceable role in alleviating drug resistance. Generally, probiotic composites are employed to assist in the eradication of *H. pylori* as well, while the key to boosting its clinical value lays clarifying the timing, dosage, and duration of probiotic addition. In conclusion, despite the achievements of anti-*H. pylori* drug delivery strategies, there are still numerous challenges for anti-*H. pylori* drug delivery strategies given the high complexity of *H. pylori* infection. In the context of the global bacterial drug resistance problem, biomaterials will certainly create more possibilities for the development and practical application of innovative antimicrobial drug delivery systems in the next few years as they are continuously tried and optimized in clinical trials.

## Author contributions

YiZ: conceptualization, methodology, and funding acquisition. CS and ZX: writing–original draft preparation. CH performed the statistical analysis. XX: screening literature. YaZ and BC: validation and software. YiZ and CH: reviewing and editing. All authors contributed to the article and approved the submitted version.

## Funding

This work was supported by the National Natural Science Foundation of China (Grant No. 82170580) and Double-Thousand Plan of Jiangxi Province (Grant No. jxsq2019201028).

## Conflict of interest

The authors declare that the research was conducted in the absence of any commercial or financial relationships that could be construed as a potential conflict of interest.

The handling editor ZG declared a past co-authorship with the author CH.

## Publisher’s note

All claims expressed in this article are solely those of the authors and do not necessarily represent those of their affiliated organizations, or those of the publisher, the editors and the reviewers. Any product that may be evaluated in this article, or claim that may be made by its manufacturer, is not guaranteed or endorsed by the publisher.

## Footnotes

^1^http://lib.ncu. http://edu.cn/

## Abbreviations

*H. pylori*: *Helicobacter pylori*
PPI: proton pump inhibitors
NPs: Nanoparticles
FRS: Floating raft system
Urel: urea transport channel protein
3SL: 3′-sialoyl lactose
p3SLP: multiple 3′-sialoyl lactose (3SL)-coupled poly (l-lysine)-based photosensitizers
SabA: acid-binding adhesin
LPs: Liposomes
LLA: linolenic acid
NLC: nanostructured lipid carriers
PEI: polyethyleneimine

## References

[ref1] Adebisi A. O. Laity P. R. Conway B. R. (2015). Formulation and evaluation of floating mucoadhesive alginate beads for targeting *Helicobacter pylori*. J. Pharm. Pharmacol. 67, 511–524. doi: 10.1111/jphp.12345, PMID: 25496042

[ref2] Amin M. Anwar F. Janjua M. R. Iqbal M. A. Rashid U. (2012). Green synthesis of silver nanoparticles through reduction with *Solanum xanthocarpum* L. berry extract: characterization, antimicrobial and urease inhibitory activities against *Helicobacter pylori*. Int. J. Mol. Sci. 13, 9923–9941. doi: 10.3390/ijms13089923, PMID: 22949839 PMC3431837

[ref3] Angsantikul P. Thamphiwatana S. Zhang Q. Spiekermann K. Zhuang J. Fang R. H.. (2018). Coating nanoparticles with gastric epithelial cell membrane for targeted antibiotic delivery against *Helicobacter pylori* infection. Adv. Therap. 1:16. doi: 10.1002/adtp.201800016, PMID: 30320205 PMC6176867

[ref4] Arangoa M. Ponchel G. Orecchioni A. Renedo M. Duchene D. Irache J. (2000). Bioadhesive potential of gliadin nanoparticulate systems. Eur. J. Pharm. Sci. 11, 333–341. doi: 10.1016/S0928-0987(00)00121-4, PMID: 11033077

[ref5] Ardekani L. S. Gargari S. L. Rasooli I. Bazl M. R. Mohammadi M. Ebrahimizadeh W.. (2013). A novel nanobody against urease activity of *Helicobacter pylori*. Int. J. Infect. Dis. 17, e723–e728. doi: 10.1016/j.ijid.2013.02.015, PMID: 23561799

[ref6] Binh T. T. Shiota S. Suzuki R. Matsuda M. Trang T. T. Kwon D. H.. (2014). Discovery of novel mutations for clarithromycin resistance in *Helicobacter pylori* by using next-generation sequencing. J. Antimicrob. Chemother. 69, 1796–1803. doi: 10.1093/jac/dku050, PMID: 24648504 PMC4054984

[ref7] Boyanova L. Hadzhiyski P. Kandilarov N. Markovska R. Mitov I. (2019). Multidrug resistance in *Helicobacter pylori*: current state and future directions. Expert. Rev. Clin. Pharmacol. 12, 909–915. doi: 10.1080/17512433.2019.1654858, PMID: 31424296

[ref8] Bugli F. Palmieri V. Torelli R. Papi M. De Spirito M. Cacaci M.. (2016). In vitro effect of clarithromycin and alginate lyase against *Helicobacter pylori* biofilm. Biotechnol. Prog. 32, 1584–1591. doi: 10.1002/btpr.2339, PMID: 27535356

[ref9] Butkovich N. Li E. Ramirez A. Burkhardt A. M. Wang S. W. (2021). Advancements in protein nanoparticle vaccine platforms to combat infectious disease. Wiley Interdiscip. Rev. Nanomed. Nanobiotechnol. 13:e1681. doi: 10.1002/wnan.1681, PMID: 33164326 PMC8052270

[ref10] Cao J. Sun Y. Berglindh T. Mellgård B. Li Z. Mårdh B.. (2000). *Helicobacter pylori*-antigen-binding fragments expressed on the filamentous M13 phage prevent bacterial growth. Biochim. Biophys. Acta 1474, 107–113. doi: 10.1016/S0304-4165(00)00005-2, PMID: 10699497

[ref11] Capurro M. I. Greenfield L. K. Prashar A. Xia S. Abdullah M. Wong H.. (2019). VacA generates a protective intracellular reservoir for *Helicobacter pylori* that is eliminated by activation of the lysosomal calcium channel TRPML1. Nat. Microbiol. 4, 1411–1423. doi: 10.1038/s41564-019-0441-6, PMID: 31110360 PMC6938649

[ref12] Chakraborti S. Bhattacharya S. Chowdhury R. Chakrabarti P. (2013). The molecular basis of inactivation of metronidazole-resistant *Helicobacter pylori* using polyethyleneimine functionalized zinc oxide nanoparticles. PLoS One 8:e70776. doi: 10.1371/journal.pone.0070776, PMID: 23951006 PMC3738536

[ref13] Chang S. H. Hsieh P. L. Tsai G. J. (2020). Chitosan inhibits *Helicobacter pylori* growth and urease production and prevents its infection of human gastric carcinoma cells. Mar. Drugs 18:542. doi: 10.3390/md18110542, PMID: 33138146 PMC7692773

[ref14] Chaves de Souza M. P. de Mattos N. H. Pedreiro L. N. Boni F. I. dos Santos Ramos M. A. Bauab T. M.. (2020). Design of Mucoadhesive Nanostructured Polyelectrolyte Complexes Based on chitosan and Hypromellose phthalate for metronidazole delivery intended to the treatment of *Helicobacter pylori* infections. Pharmaceutics 12:1211. doi: 10.3390/pharmaceutics12121211, PMID: 33327588 PMC7765050

[ref15] Chen Y. Li Y. Guo L. Hong J. Zhao W. Hu X.. (2020). Bibliometric analysis of the Inflammasome and Pyroptosis in brain. Front. Pharmacol. 11:626502. doi: 10.3389/fphar.2020.626502 33551822 PMC7854385

[ref16] Chen X. Li P. Shen Y. Zou Y. Yuan G. Hu H. (2019). Rhamnolipid-involved antibiotics combinations improve the eradication of *Helicobacter pylori* biofilm in vitro: a comparison with conventional triple therapy. Microb. Pathog. 131, 112–119. doi: 10.1016/j.micpath.2019.04.001, PMID: 30951818

[ref17] Chen X.-n. Shen Y.-n. Li P.-y. Zou Y.-q. Hu H.-y. (2018). Bacterial biofilms: characteristics and combat strategies. Acta Pharm. Sin. 12, 2040–2049. doi: 10.16438/J.05134870.2018-0892

[ref18] Chen Q. Wang C. Zhang X. Chen G. Hu Q. Li H.. (2019). *In situ* sprayed bioresponsive immunotherapeutic gel for post-surgical cancer treatment. Nat. Nanotechnol. 14, 89–97. doi: 10.1038/s41565-018-0319-4, PMID: 30531990

[ref19] Chen L. Xu W. Lee A. He J. Huang B. Zheng W.. (2018). The impact of *Helicobacter pylori* infection, eradication therapy and probiotic supplementation on gut microenvironment homeostasis: an open-label, randomized clinical trial. EBioMedicine 35, 87–96. doi: 10.1016/j.ebiom.2018.08.028, PMID: 30145102 PMC6161473

[ref20] Chmiela M. Kupcinskas J. (2019). Review: pathogenesis of *Helicobacter pylori* infection. Helicobacter 24:e12638. doi: 10.1111/hel.12638 31486234 PMC6771490

[ref21] Coker O. O. Dai Z. Nie Y. Zhao G. Cao L. Nakatsu G.. (2018). Mucosal microbiome dysbiosis in gastric carcinogenesis. Gut 67, 1024–1032. doi: 10.1136/gutjnl-2017-314281, PMID: 28765474 PMC5969346

[ref22] Cong Y. Geng J. Wang H. Su J. Arif M. Dong Q.. (2019). Ureido-modified carboxymethyl chitosan-graft-stearic acid polymeric nano-micelles as a targeted delivering carrier of clarithromycin for *Helicobacter pylori*: preparation and in vitro evaluation. Int. J. Biol. Macromol. 129, 686–692. doi: 10.1016/j.ijbiomac.2019.01.227, PMID: 30772413

[ref23] Cui Y. Zhou K. Strugatsky D. Wen Y. Sachs G. Zhou Z. H.. (2019). pH-dependent gating mechanism of the *Helicobacter pylori* urea channel revealed by cryo-EM. Sci. Adv. 5:8423. doi: 10.1126/sciadv.aav8423 PMC6426461 30906870

[ref24] Dai T. Huang Y. Y. Hamblin M. R. (2009). Photodynamic therapy for localized infections—state of the art. Photodiagn. Photodyn. Ther. 6, 170–188. doi: 10.1016/j.pdpdt.2009.10.008, PMID: 19932449 PMC2811240

[ref25] Darroudi M. Gholami M. Rezayi M. Khazaei M. (2021). An overview and bibliometric analysis on the colorectal cancer therapy by magnetic functionalized nanoparticles for the responsive and targeted drug delivery. J. Nanobiotechnol. 19:399. doi: 10.1186/s12951-021-01150-6, PMID: 34844632 PMC8630862

[ref26] de Ávila B. E. Angsantikul P. Li J. Angel Lopez-Ramirez M. Ramírez-Herrera D. E. Thamphiwatana S.. (2017). Micromotor-enabled active drug delivery for in vivo treatment of stomach infection. Nat. Commun. 8:272. doi: 10.1038/s41467-017-00309-w, PMID: 28814725 PMC5559609

[ref27] de Reuse H. Vinella D. Cavazza C. (2013). Common themes and unique proteins for the uptake and trafficking of nickel, a metal essential for the virulence of *Helicobacter pylori*. Front. Cell. Infect. Microbiol. 3:94. doi: 10.3389/fcimb.2013.00094 24367767 PMC3856676

[ref28] de Souza M. P. C. de Camargo B. A. F. Spósito L. Fortunato G. C. Carvalho G. C. Marena G. D.. (2021). Highlighting the use of micro and nanoparticles based-drug delivery systems for the treatment of *Helicobacter pylori* infections. Crit. Rev. Microbiol. 47, 435–460. doi: 10.1080/1040841X.2021.1895721, PMID: 33725462

[ref29] Demidova T. N. Hamblin M. R. (2004). Photodynamic therapy targeted to pathogens. Int. J. Immunopathol. Pharmacol. 17, 245–254. doi: 10.1177/039463200401700304, PMID: 15461858 PMC3071683

[ref30] Fallone C. A. Chiba N. van Zanten S. V. Fischbach L. Gisbert J. P. Hunt R. H.. (2016). The Toronto consensus for the treatment of *Helicobacter pylori* infection in adults. Gastroenterology 151, 51–69.e14. doi: 10.1053/j.gastro.2016.04.006, PMID: 27102658

[ref31] Ferreira R. Sousa C. Gonçalves R. F. S. Pinheiro A. C. Oleastro M. Wagemans J.. (2022). Characterization and genomic analysis of a new phage infecting *Helicobacter pylori*. Int. J. Mol. Sci. 23:885. doi: 10.3390/ijms23147885, PMID: 35887231 PMC9319048

[ref32] Fulgione A. Nocerino N. Iannaccone M. Roperto S. Capuano F. Roveri N.. (2016). Lactoferrin adsorbed onto biomimetic hydroxyapatite Nanocrystals controlling–*in vivo*–the *Helicobacter pylori* infection. PLoS One 11:e0158646. doi: 10.1371/journal.pone.0158646, PMID: 27384186 PMC4934871

[ref33] Garcez A. S. Nuñez S. C. Hamblim M. R. Suzuki H. Ribeiro M. S. (2010). Photodynamic therapy associated with conventional endodontic treatment in patients with antibiotic-resistant microflora: a preliminary report. J. Endod. 36, 1463–1466. doi: 10.1016/j.joen.2010.06.001, PMID: 20728710

[ref34] Garcia T. Lafuente D. Blanco J. Sánchez D. J. Sirvent J. J. Domingo J. L.. (2016). Oral subchronic exposure to silver nanoparticles in rats. Food Chem. Toxicol. 92, 177–187. doi: 10.1016/j.fct.2016.04.010, PMID: 27090107

[ref35] Gonçalves I. C. Henriques P. C. Seabra C. L. Martins M. C. (2014). The potential utility of chitosan micro/nanoparticles in the treatment of gastric infection. Expert Rev. Anti-Infect. Ther. 12, 981–992. doi: 10.1586/14787210.2014.930663, PMID: 24981812

[ref36] Gong Y. Yuan Y. (2018). Resistance mechanisms of *Helicobacter pylori* and its dual target precise therapy. Crit. Rev. Microbiol. 44, 371–392. doi: 10.1080/1040841X.2017.1418285, PMID: 29293032

[ref37] Gopalakrishnan V. Masanam E. Ramkumar V. S. Baskaraligam V. Selvaraj G. (2020). Influence of N-acylhomoserine lactonase silver nanoparticles on the quorum sensing system of *Helicobacter pylori*: a potential strategy to combat biofilm formation. J. Basic Microbiol. 60, 207–215. doi: 10.1002/jobm.201900537, PMID: 31960983

[ref38] Graham D. Y. (2014). History of *Helicobacter pylori*, duodenal ulcer, gastric ulcer and gastric cancer. World J. Gastroenterol. 20, 5191–5204. doi: 10.3748/wjg.v20.i18.5191, PMID: 24833849 PMC4017034

[ref39] Graham D. Y. Dore M. P. (2016). *Helicobacter pylori* therapy: a paradigm shift. Expert Rev. Anti-Infect. Ther. 14, 577–585. doi: 10.1080/14787210.2016.1178065, PMID: 27077447 PMC4939773

[ref40] Gurunathan S. Jeong J. K. Han J. W. Zhang X. F. Park J. H. Kim J. H. (2015). Multidimensional effects of biologically synthesized silver nanoparticles in *Helicobacter pylori*, *Helicobacter felis*, and human lung (L132) and lung carcinoma A549 cells. Nanoscale Res. Lett. 10:35. doi: 10.1186/s11671-015-0747-0, PMID: 25852332 PMC4384991

[ref41] Häfeli U. O. (2004). Magnetically modulated therapeutic systems. Int. J. Pharm. 277, 19–24. doi: 10.1016/j.ijpharm.2003.03.002 15158965

[ref42] Han X. Alu A. Liu H. Shi Y. Wei X. Cai L.. (2022). Biomaterial-assisted biotherapy: a brief review of biomaterials used in drug delivery, vaccine development, gene therapy, and stem cell therapy. Bioact. Mat. 17, 29–48. doi: 10.1016/j.bioactmat.2022.01.011, PMID: 35386442 PMC8958282

[ref43] Hathroubi S. Servetas S. L. Windham I. Merrell D. S. Ottemann K. M. (2018). *Helicobacter pylori* biofilm formation and its potential role in pathogenesis. Microbiol. Mol. Biol. Rev. 82:18. doi: 10.1128/MMBR.00001-18, PMID: 29743338 PMC5968456

[ref44] Heidelbaugh J. J. (2013). Proton pump inhibitors and risk of vitamin and mineral deficiency: evidence and clinical implications. Therap. Adv. Drug Safety 4, 125–133. doi: 10.1177/2042098613482484, PMID: 25083257 PMC4110863

[ref45] Hejazi R. Amiji M. (2003). Chitosan-based gastrointestinal delivery systems. J. Control. Release 89, 151–165. doi: 10.1016/S0168-3659(03)00126-3 12711440

[ref46] Hooi J. K. Y. Lai W. Y. Ng W. K. Suen M. M. Y. Underwood F. E. Tanyingoh D.. (2017). Global prevalence of *Helicobacter pylori* infection: systematic review and meta-analysis. Gastroenterology 153, 420–429. doi: 10.1053/j.gastro.2017.04.022 28456631

[ref47] Huang L. Xuan Y. Koide Y. Zhiyentayev T. Tanaka M. Hamblin M. R. (2012). Type I and type II mechanisms of antimicrobial photodynamic therapy: an in vitro study on gram-negative and gram-positive bacteria. Lasers Surg. Med. 44, 490–499. doi: 10.1002/lsm.22045, PMID: 22760848 PMC3428129

[ref48] Hussain Z. Arooj M. Malik A. Hussain F. Safdar H. Khan S.. (2018). Nanomedicines as emerging platform for simultaneous delivery of cancer therapeutics: new developments in overcoming drug resistance and optimizing anticancer efficacy. Artif. Cells Nanomed. Biotechnol. 46, 1015–1024. doi: 10.1080/21691401.2018.1478420, PMID: 29873531

[ref49] Im B. N. Shin H. Lim B. Lee J. Kim K. S. Park J. M.. (2021). *Helicobacter pylori*-targeting multiligand photosensitizer for effective antibacterial endoscopic photodynamic therapy. Biomaterials 271:120745. doi: 10.1016/j.biomaterials.2021.120745, PMID: 33740616

[ref50] Jeong S. Park W. Lee C. S. Na K. (2014). A cancer-recognizing polymeric photosensitizer based on the tumor extracellular pH response of conjugated polymers for targeted cancer photodynamic therapy. Macromol. Biosci. 14, 1688–1695. doi: 10.1002/mabi.201400361, PMID: 25251581

[ref51] Jing Z. W. Jia Y. Y. Wan N. Luo M. Huan M. L. Kang T. B.. (2016). Design and evaluation of novel pH-sensitive ureido-conjugated chitosan/TPP nanoparticles targeted to *Helicobacter pylori*. Biomaterials 84, 276–285. doi: 10.1016/j.biomaterials.2016.01.045, PMID: 26851392

[ref52] Kao J. Y. Zhang M. Miller M. J. Mills J. C. Wang B. Liu M.. (2010). *Helicobacter pylori* immune escape is mediated by dendritic cell-induced Treg skewing and Th17 suppression in mice. Gastroenterology 138, 1046–1054. doi: 10.1053/j.gastro.2009.11.043, PMID: 19931266 PMC2831148

[ref53] Khan S. Sharaf M. Ahmed I. Khan T. U. Shabana S. Arif M.. (2022). Potential utility of nano-based treatment approaches to address the risk of *Helicobacter pylori*. Expert Rev. Anti-Infect. Ther. 20, 407–424. doi: 10.1080/14787210.2022.1990041, PMID: 34658307

[ref54] Kim Y. S. Song M. Y. Park J. D. Song K. S. Ryu H. R. Chung Y. H.. (2010). Subchronic oral toxicity of silver nanoparticles. Part. Fibre Toxicol. 7:20. doi: 10.1186/1743-8977-7-20, PMID: 20691052 PMC2928176

[ref55] Lang X. Wang T. Sun M. Chen X. Liu Y. (2020). Advances and applications of chitosan-based nanomaterials as oral delivery carriers: a review. Int. J. Biol. Macromol. 154, 433–445. doi: 10.1016/j.ijbiomac.2020.03.148, PMID: 32194103

[ref56] Langer R. (1990). New methods of drug delivery. Science 249, 1527–1533. doi: 10.1126/science.2218494 2218494

[ref57] Lau C. S. Ward A. Chamberlain R. S. (2016). Probiotics improve the efficacy of standard triple therapy in the eradication of *Helicobacter pylori*: a meta-analysis. Infect. Drug Resist. 9, 275–289. doi: 10.2147/IDR.S117886, PMID: 27994474 PMC5153259

[ref58] Lin C. C. Huang W. C. Su C. H. Lin W. D. Wu W. T. Yu B.. (2020). Effects of multi-strain probiotics on immune responses and metabolic balance in *Helicobacter pylori*-infected mice. Nutrients 12:2476. doi: 10.3390/nu12082476, PMID: 32824501 PMC7468736

[ref59] Lin Y. H. Lu K. Y. Tseng C. L. Wu J. Y. Chen C. H. Mi F. L. (2017). Development of genipin-crosslinked fucoidan/chitosan-N-arginine nanogels for preventing helicobacter infection. Nanomedicine 12, 1491–1510. doi: 10.2217/nnm-2017-0055, PMID: 28524785

[ref60] Ling D. Bae B. C. Park W. Na K. (2012). Photodynamic efficacy of photosensitizers under an attenuated light dose via lipid nano-carrier-mediated nuclear targeting. Biomaterials 33, 5478–5486. doi: 10.1016/j.biomaterials.2012.04.023, PMID: 22560197

[ref61] Lionetti E. Indrio F. Pavone L. Borrelli G. Cavallo L. Francavilla R. (2010). Role of probiotics in pediatric patients with *Helicobacter pylori* infection: a comprehensive review of the literature. Helicobacter 15, 79–87. doi: 10.1111/j.1523-5378.2009.00743.x, PMID: 20402810

[ref62] Luo M. Jia Y. Y. Jing Z. W. Li C. Zhou S. Y. Mei Q. B.. (2018). Construction and optimization of pH-sensitive nanoparticle delivery system containing PLGA and UCCs-2 for targeted treatment of *Helicobacter pylori*. Colloids Surf. B Biointerfaces 164, 11–19. doi: 10.1016/j.colsurfb.2018.01.008, PMID: 29367052

[ref63] Malfertheiner P. Megraud F. O’Morain C. Bazzoli F. el-Omar E. Graham D.. (2007). Current concepts in the management of *Helicobacter pylori* infection: the Maastricht III consensus report. Gut 56, 772–781. doi: 10.1136/gut.2006.101634, PMID: 17170018 PMC1954853

[ref64] Malfertheiner P. Megraud F. O’Morain C. A. Gisbert J. P. Kuipers E. J. Axon A. T.. (2017). Management of *Helicobacter pylori* infection-the Maastricht V/Florence consensus report. Gut 66, 6–30. doi: 10.1136/gutjnl-2016-312288, PMID: 27707777

[ref65] Mera R. M. Bravo L. E. Camargo M. C. Bravo J. C. Delgado A. G. Romero-Gallo J.. (2018). Dynamics of *Helicobacter pylori* infection as a determinant of progression of gastric precancerous lesions: 16-year follow-up of an eradication trial. Gut 67, 1239–1246. doi: 10.1136/gutjnl-2016-311685, PMID: 28647684 PMC5742304

[ref66] Muñoz A. B. Stepanian J. Trespalacios A. A. Vale F. F. (2020). Bacteriophages of *Helicobacter pylori*. Front. Microbiol. 11:549084. doi: 10.3389/fmicb.2020.549084, PMID: 33281754 PMC7688985

[ref67] Nocerino N. Fulgione A. Iannaccone M. Tomasetta L. Ianniello F. Martora F.. (2014). Biological activity of lactoferrin-functionalized biomimetic hydroxyapatite nanocrystals. Int. J. Nanomedicine 9, 1175–1184. doi: 10.2147/IJN.S55060, PMID: 24623976 PMC3949719

[ref68] Nogueira F. Gonçalves I. C. Martins M. C. (2013). Effect of gastric environment on *Helicobacter pylori* adhesion to a mucoadhesive polymer. Acta Biomater. 9, 5208–5215. doi: 10.1016/j.actbio.2012.09.011, PMID: 22995406

[ref69] Ouyang Y. Zhu Z. Huang L. Zeng C. Zhang L. Wu W. K.. (2021). Research trends on clinical *Helicobacter pylori* eradication: a Bibliometric analysis from 1983 to 2020. Helicobacter 26:e12835. doi: 10.1111/hel.12835, PMID: 34258827

[ref70] Palechor-Trochez J. J. Ramírez-Gonzales G. Villada-Castillo H. S. Solanilla-Duque J. F. (2021). A review of trends in the development of bionanocomposites from lignocellulosic and polyacids biomolecules as packing material making alternative: a bibliometric analysis. Int. J. Biol. Macromol. 192, 832–868. doi: 10.1016/j.ijbiomac.2021.10.003, PMID: 34634331

[ref71] Park H. Lee J. Jeong S. Im B. N. Kim M. K. Yang S. G.. (2016). Lipase-sensitive Transfersomes based on photosensitizer/Polymerizable lipid conjugate for selective antimicrobial photodynamic therapy of acne. Adv. Healthc. Mater. 5, 3139–3147. doi: 10.1002/adhm.201600815, PMID: 27863184

[ref72] Portero A. Remuñán-López C. Criado M. T. Alonso M. J. (2002). Reacetylated chitosan microspheres for controlled delivery of anti-microbial agents to the gastric mucosa. J. Microencapsul. 19, 797–809. doi: 10.1080/0265204021000022761, PMID: 12569028

[ref73] Pouwels S. Lalmohamed A. Souverein P. Cooper C. Veldt B. J. Leufkens H. G.. (2011). Use of proton pump inhibitors and risk of hip/femur fracture: a population-based case-control study. Osteoporosis Int. 22, 903–910. doi: 10.1007/s00198-010-1337-8, PMID: 20585937 PMC3034906

[ref74] Praditya D. Kirchhoff L. Brüning J. Rachmawati H. Steinmann J. Steinmann E. (2019). Anti-infective properties of the Golden spice Curcumin. Front. Microbiol. 10:912. doi: 10.3389/fmicb.2019.00912, PMID: 31130924 PMC6509173

[ref75] Qu J. Zhao X. Liang Y. Zhang T. Ma P. X. Guo B. (2018). Antibacterial adhesive injectable hydrogels with rapid self-healing, extensibility and compressibility as wound dressing for joints skin wound healing. Biomaterials 183, 185–199. doi: 10.1016/j.biomaterials.2018.08.044, PMID: 30172244

[ref76] Rajinikanth P. S. Balasubramaniam J. Mishra B. (2007). Development and evaluation of a novel floating in situ gelling system of amoxicillin for eradication of *Helicobacter pylori*. Int. J. Pharm. 335, 114–122. doi: 10.1016/j.ijpharm.2006.11.008, PMID: 17141986

[ref77] Reshetnyak V. I. Reshetnyak T. M. (2017). Significance of dormant forms of *Helicobacter pylori* in ulcerogenesis. World J. Gastroenterol. 23, 4867–4878. doi: 10.3748/wjg.v23.i27.4867, PMID: 28785141 PMC5526757

[ref78] Sachin K. Karn S. K. (2021). Microbial fabricated Nanosystems: applications in drug delivery and targeting. Front. Chem. 9:617353. doi: 10.3389/fchem.2021.617353, PMID: 33959586 PMC8093762

[ref79] Saha A. Hammond C. E. Beeson C. Peek R. M. Jr. Smolka A. J. (2010). *Helicobacter pylori* represses proton pump expression and inhibits acid secretion in human gastric mucosa. Gut 59, 874–881. doi: 10.1136/gut.2009.194795, PMID: 20581234 PMC2980826

[ref80] Sánchez S. Soler L. Katuri J. (2015). Chemically powered micro- and nanomotors. Angew. Chem. Int. Ed. Engl. 54, 1414–1444. doi: 10.1002/anie.201406096 25504117

[ref81] Seabra C. L. Nunes C. Brás M. Gomez-Lazaro M. Reis C. A. Gonçalves I. C.. (2018). Lipid nanoparticles to counteract gastric infection without affecting gut microbiota. Eur. J. Pharm. Biopharm. 127, 378–386. doi: 10.1016/j.ejpb.2018.02.030, PMID: 29524597

[ref82] Silva É. L. Carvalho J. F. Pontes T. R. Oliveira E. E. Francelino B. L. Medeiros A. C.. (2009). Development of a magnetic system for the treatment of *Helicobacter pylori* infections. J. Magn. Magn. Mater. 321, 1566–1570. doi: 10.1016/j.jmmm.2009.02.087

[ref83] Sreelakshmi C. Datta K. K. Yadav J. S. Reddy B. V. (2011). Honey derivatized au and ag nanoparticles and evaluation of its antimicrobial activity. J. Nanosci. Nanotechnol. 11, 6995–7000. doi: 10.1166/jnn.2011.4240, PMID: 22103111

[ref84] Su Y.-R. Yu S.-H. Chao A.-C. Wu J.-Y. Lin Y.-F. Lu K.-Y.. (2016). Preparation and properties of pH-responsive, self-assembled colloidal nanoparticles from guanidine-containing polypeptide and chitosan for antibiotic delivery. Colloids Surf. A Physicochem. Eng. Asp. 494, 9–20. doi: 10.1016/j.colsurfa.2016.01.017

[ref85] Suerbaum S. Smith J. M. Bapumia K. Morelli G. Smith N. H. Kunstmann E.. (1998). Free recombination within *Helicobacter pylori*. Proc. Natl. Acad. Sci. U. S. A. 95, 12619–12624. doi: 10.1073/pnas.95.21.12619, PMID: 9770535 PMC22880

[ref86] Thamphiwatana S. Fu V. Zhu J. Lu D. Gao W. Zhang L. (2013). Nanoparticle-stabilized liposomes for pH-responsive gastric drug delivery. Langmuir 29, 12228–12233. doi: 10.1021/la402695c, PMID: 23987129 PMC4059759

[ref87] Thombre N. A. Gide P. S. (2016). Floating-bioadhesive gastroretentive Caesalpinia pulcherrima-based beads of amoxicillin trihydrate for *Helicobacter pylori* eradication. Drug Deliv. 23, 405–419. doi: 10.3109/10717544.2014.916766, PMID: 24870198

[ref88] Tshibangu-Kabamba E. Yamaoka Y. (2021). *Helicobacter pylori* infection and antibiotic resistance–from biology to clinical implications. Nat. Rev. Gastroenterol. Hepatol. 18, 613–629. doi: 10.1038/s41575-021-00449-x, PMID: 34002081

[ref89] Vázquez E. Villaverde A. (2013). Microbial biofabrication for nanomedicine: biomaterials, nanoparticles and beyond. Nanomedicine 8, 1895–1898. doi: 10.2217/nnm.13.164, PMID: 24279484

[ref90] Walker D. Käsdorf B. T. Jeong H. H. Lieleg O. Fischer P. (2015). Enzymatically active biomimetic micropropellers for the penetration of mucin gels. Sci. Adv. 1:e1500501. doi: 10.1126/sciadv.1500501, PMID: 26824056 PMC4730841

[ref91] Wang M. Wang C. Chen M. Xi Y. Cheng W. Mao C.. (2019). Efficient angiogenesis-based diabetic wound healing/skin reconstruction through bioactive antibacterial adhesive ultraviolet shielding Nanodressing with exosome release. ACS Nano 13, 10279–10293. doi: 10.1021/acsnano.9b03656, PMID: 31483606

[ref92] Watanabe Y. Kim H. S. Castoro R. J. Chung W. Estecio M. R. Kondo K.. (2009). Sensitive and specific detection of early gastric cancer with DNA methylation analysis of gastric washes. Gastroenterology 136, 2149–2158. doi: 10.1053/j.gastro.2009.02.085, PMID: 19375421 PMC2722957

[ref93] Wu Y. Song Z. Deng G. Jiang K. Wang H. Zhang X.. (2021). Gastric acid powered Nanomotors release antibiotics for in vivo treatment of *Helicobacter pylori* infection. Small 17:e2006877. doi: 10.1002/smll.202006877, PMID: 33619851

[ref94] Wu T. Wang L. Gong M. Lin Y. Xu Y. Ye L.. (2019). Synergistic effects of nanoparticle heating and amoxicillin on *H. pylori* inhibition. J. Magn. Magn. Mater. 485, 95–104. doi: 10.1016/j.jmmm.2019.04.076

[ref95] Yan L. X. Wang B. B. Zhao X. Chen L. J. Yan X. P. (2021). A pH-responsive persistent luminescence Nanozyme for selective imaging and killing of *Helicobacter pylori* and common resistant bacteria. ACS Appl. Mater. Interfaces 13, 60955–60965. doi: 10.1021/acsami.1c21318, PMID: 34904434

[ref96] Yang S. J. Huang C. H. Yang J. C. Wang C. H. Shieh M. J. (2020). Residence time-extended nanoparticles by magnetic field improve the eradication efficiency of *Helicobacter pylori*. ACS Appl. Mater. Interfaces 12, 54316–54327. doi: 10.1021/acsami.0c13101, PMID: 33236884

[ref97] Yang J. C. Lu C. W. Lin C. J. (2014). Treatment of *Helicobacter pylori* infection: current status and future concepts. World J. Gastroenterol. 20, 5283–5293. doi: 10.3748/wjg.v20.i18.5283, PMID: 24833858 PMC4017043

[ref98] Yun Y. H. Lee B. K. Park K. (2015). Controlled drug delivery: historical perspective for the next generation. J. Control. Release 219, 2–7. doi: 10.1016/j.jconrel.2015.10.005, PMID: 26456749 PMC4656096

[ref99] Zaidi S. Misba L. Khan A. U. (2017). Nano-therapeutics: a revolution in infection control in post antibiotic era. Nanomedicine 13, 2281–2301. doi: 10.1016/j.nano.2017.06.015, PMID: 28673854

[ref100] Zhang Y. Chen Y. Lo C. Zhuang J. Angsantikul P. Zhang Q.. (2019). Inhibition of pathogen adhesion by bacterial outer membrane-coated nanoparticles. Angew. Chem. Int. Ed. Engl. 58, 11404–11408. doi: 10.1002/anie.201906280, PMID: 31206942

[ref101] Zhang Y. Li H. Wang Q. Hao X. Li H. Sun H.. (2018). Rationally designed self-assembling nanoparticles to overcome mucus and epithelium transport barriers for oral vaccines against *Helicobacter pylori*. Adv. Funct. Mater. 28:1802675. doi: 10.1002/adfm.201802675

[ref102] Zhang N. Mei K. Guan P. Hu X. Zhao Y. (2020). Protein-based artificial Nanosystems in cancer therapy. Small 16:e1907256. doi: 10.1002/smll.201907256, PMID: 32378796

[ref103] Zhang T. Zhang B. Tian W. Ma X. Wang F. Wang P.. (2022). A Bibliometric analysis of atrophic gastritis from 2011 to 2021. Front. Med. 9:843395. doi: 10.3389/fmed.2022.843395, PMID: 35252276 PMC8891522

[ref104] Zhang W. Zhou Y. Fan Y. Cao R. Xu Y. Weng Z.. (2022). Metal-organic-framework-based hydrogen-release platform for multieffective *Helicobacter pylori* targeting therapy and intestinal Flora protective capabilities. Adv. Mater. 34:e2105738. doi: 10.1002/adma.202105738, PMID: 34655499

[ref105] Zhi X. Liu Y. Lin L. Yang M. Zhang L. Zhang L.. (2019). Oral pH sensitive GNS@ab nanoprobes for targeted therapy of *Helicobacter pylori* without disturbance gut microbiome. Nanomedicine 20:102019. doi: 10.1016/j.nano.2019.102019, PMID: 31125676

[ref106] Zhu S. Liu Y. Gu Z. Zhao Y. (2021). A Bibliometric analysis of advanced healthcare materials: research trends of biomaterials in healthcare application. Adv. Healthc. Mater. 10:e2002222. doi: 10.1002/adhm.202002222, PMID: 33599117

